# Interactions of a Novel Anthracycline with Oligonucleotide DNA and Cyclodextrins in an Aqueous Environment

**DOI:** 10.1021/acs.jpcb.4c02213

**Published:** 2024-06-20

**Authors:** Georgios Mikaelian, Grigorios Megariotis, Doros N. Theodorou

**Affiliations:** † School of Chemical Engineering, National Technical University of Athens (NTUA), 9 Heroon Polytechniou Street, Zografou Campus, 15780 Athens, GR ,Greece; ‡ School of Engineering, Department of Mineral Resources Engineering, University of Western Macedonia, 50100 Kozani, Greece

## Abstract

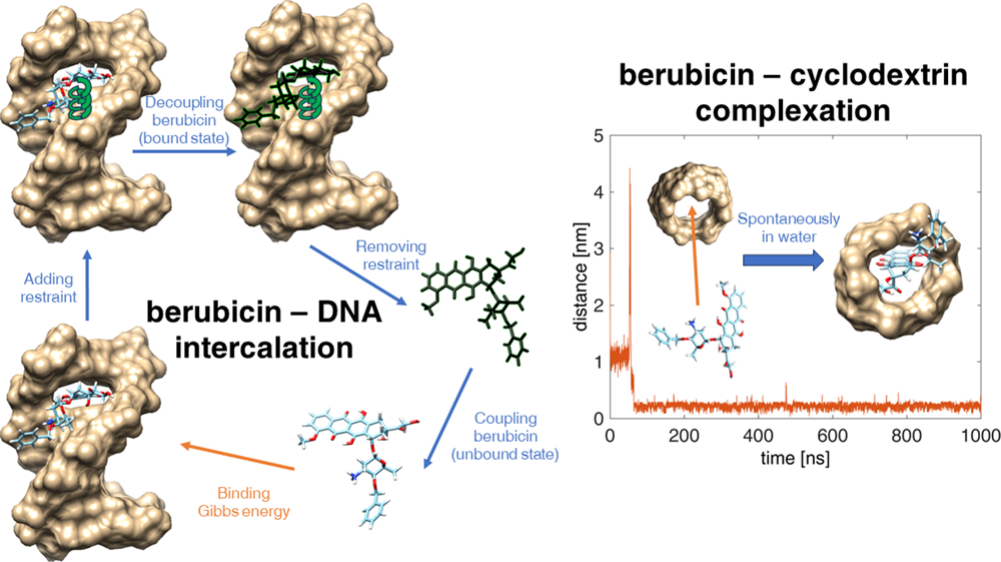

Berubicin, a chemotherapy medication belonging to the class of anthracyclines, is simulated in double-stranded DNA sequences and cyclodextrins in an aqueous environment via full-atom molecular dynamics simulations on the time scale of microseconds. The drug is studied in both the neutral and protonated states so as to better comprehend the role of its charge in the formed complexes. The noncovalent berubicin–DNA and berubicin–cyclodextrin complexes are investigated in detail, paying special attention to their thermodynamic description by employing the double decoupling method, the solvent balance method, the weighted solvent accessible surface model, and the linear interaction energy method. A novel approach for extracting the desolvation thermodynamics of the binding process is also presented. Both the binding and desolvation Gibbs energies are decomposed into entropic and enthalpic contributions so as to elucidate the nature of complexation and its driving forces. Selected structural and geometrical properties of all the complexes, which are all stable, are analyzed. Both cyclodextrins under consideration are widely utilized for drug delivery purposes, and a comparative investigation between their bound states with berubicin is carried out.

## 1 Introduction

**Figure 1:**
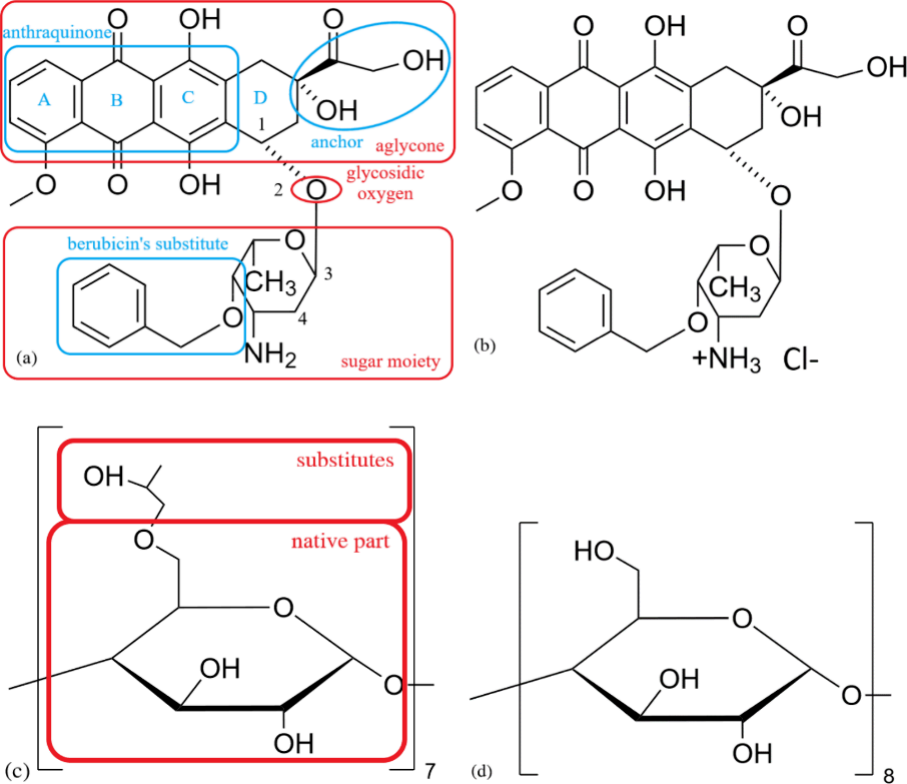
Molecules studied in this article: (a) neutral berubicin, (b) protonated berubicin, (c) repeating unit of HP-β-CD, and (d) repeating unit of γ-CD.

Berubicin is a chemotherapy medication that has primarily been developed for the treatment of glioblastoma, the most aggressive and treatment-resistant type of brain cancer.^1−4^ This drug belongs to the family of anthracyclines^1,2,5^ and is able to cross the blood–brain barrier and reach brain tumors effectively.^1,6^ Anthracyclines aim mainly at inducing DNA damage in the targeted tumor cells by interfering with the action of topoisomerase II, a nuclear enzyme that fixes topological problems in DNA caused mainly by the processes of replication and transcription.^2,5−7^ Intercalation into DNA is numbered among the mechanisms of action of anthracyclines due to the existence of an aglycone planar structure that is capable of intercalating between the strands of DNA.^7,8^ Aglycone is a system of four rings with one of them being a saturated and substituted ring to which a side chain and one or more sugar residues are covalently attached (see Figure 1a);^7,8^ it is noted that the characteristic reddish color of anthracyclines is owed to this system of rings.^8,9^ Berubicin is an O-benzylated derivative of doxorubicin, one of the prototype molecules of the anthracycline class; the difference between them lies only in the sugar moiety. The advantage of berubicin in relation to other anthracyclines is its ability to circumvent the multidrug resistance mechanisms,^10−13^ in contrast with the other anthracyclines.^10,14^ This makes it more effective at inducing DNA damage, while at the same time being less lethal, compared to the parental drug, doxorubicin.^1^ In addition to the advantages, substituting berubicin for doxorubicin makes the drug more hydrophobic which may be undesired in certain cases. As is discussed later, however, this side effect could be suppressed with the use of cyclodextrins.

This article focuses on berubicin, but, when necessary, the presented results are discussed in view of findings from ref (15) concerning doxorubicin or other published articles focusing on either doxorubicin or other anthracyclines. The approach adopted herein is solely *in silico* and more specifically is based on full-atom molecular dynamics (MD) simulations on the time scale of microseconds; this classical simulation method tracks the time evolution of a system of interacting particles described by a force field or a combination of force fields and may be envisioned as a “computational microscope” shedding light on the structure, dynamics, and thermodynamics of complex chemical and biomolecular systems.^16^ Moreover, in many cases, MD provides information that experimentally is either not easily accessible or not accessible at all.^17−19^ At this point, it should be noted that, to the best of our knowledge, there do not exist published detailed computational studies regarding berubicin at the time of writing of this article. In view of the fact that the anthracycline considered herein is a very promising chemotherapy medication with unique properties, we present a detailed study of this drug in different biological environments in order to better comprehend how it behaves from a nanoscopic point of view. To be more specific, first, the interactions of berubicin with DNA in an aqueous phase are investigated paying special attention to the detailed thermodynamic description of these systems. For the calculation of the binding Gibbs energies from the MD simulations, well-established methods are employed such as the double decoupling method (DDM) and the solvent balance method (SBM). As far as the formation of berubicin–DNA complexes is concerned, these are formed by docking experiments and, after implementation of a proper equilibration stage, they are simulated in an aqueous environment at a reasonable salt concentration by long full-atom MD simulations on the time scale of microseconds.

In addition to DNA, berubicin is simulated in water solutions containing cyclodextrins which are well-known vehicles for drug delivery purposes. Cyclodextrins are cyclic oligosaccharides that have the shape of a truncated cone consisting of an interior hydrophobic cavity and an exterior hydrophilic surface. The formed complexes between cyclodextrins and drugs (or other small molecules) are of noncovalent nature, and this complexation may modify/improve the properties of the guest molecule, such as its aqueous solubility and bioavailability.^20−23^ In pharmaceutical applications, cyclodextrins may be employed as excipients, or even as active pharmaceutical ingredients, in formulations for oral, parenteral, or other administration. It is also noteworthy that at least 100 pharmaceutical drugs containing cyclodextrins are found in the market.^24^ Another aim of this study is to compare the effect of different cyclodextrins on the properties of the created supramolecular complexes. Anthracyclines manifest some disadvantages which may be alleviated by complexation with cyclodextrins. For instance, in the related literature, the anthraquinone group has been associated with photoreactivity,^25−29^ which results in the anthracycline molecule being less stable in water. In addition, it is known that the anthracyclines undergo dimerization,^27,30−32^ which has as a consequence the reduction of their anticancer action. A major adverse effect is their cardiotoxicity, which is believed to be induced through certain chemical paths.^33,34^ So, we could benefit from a cyclodextrin complexation since the drug studied herein is highly hydrophobic. Again, as in the case of berubicin–DNA complexes, particular attention is paid to the thermodynamic description of the berubicin–cyclodextrin complexes by applying the linear interaction energy (LIE) method that has been successfully employed in published computational studies concerning other drug–cyclodextrin complexes.^15,35,36^

This article is organized as follows. All the biomolecular systems under consideration herein and the employed *in silico* methods are given in full detail in Section 2. Next, in Section 3, we present the simulation results along with a thorough discussion providing special emphasis on the thermodynamic description of the considered systems. More specifically, the first part of Section 3 concerns the berubicin–DNA complexes, while the rest of the section focuses on the berubicin–cyclodextrin complexes. Section 4 is devoted to the main conclusions extracted from the research presented in this article.

## 2 Models and Methods

**Figure 2:**
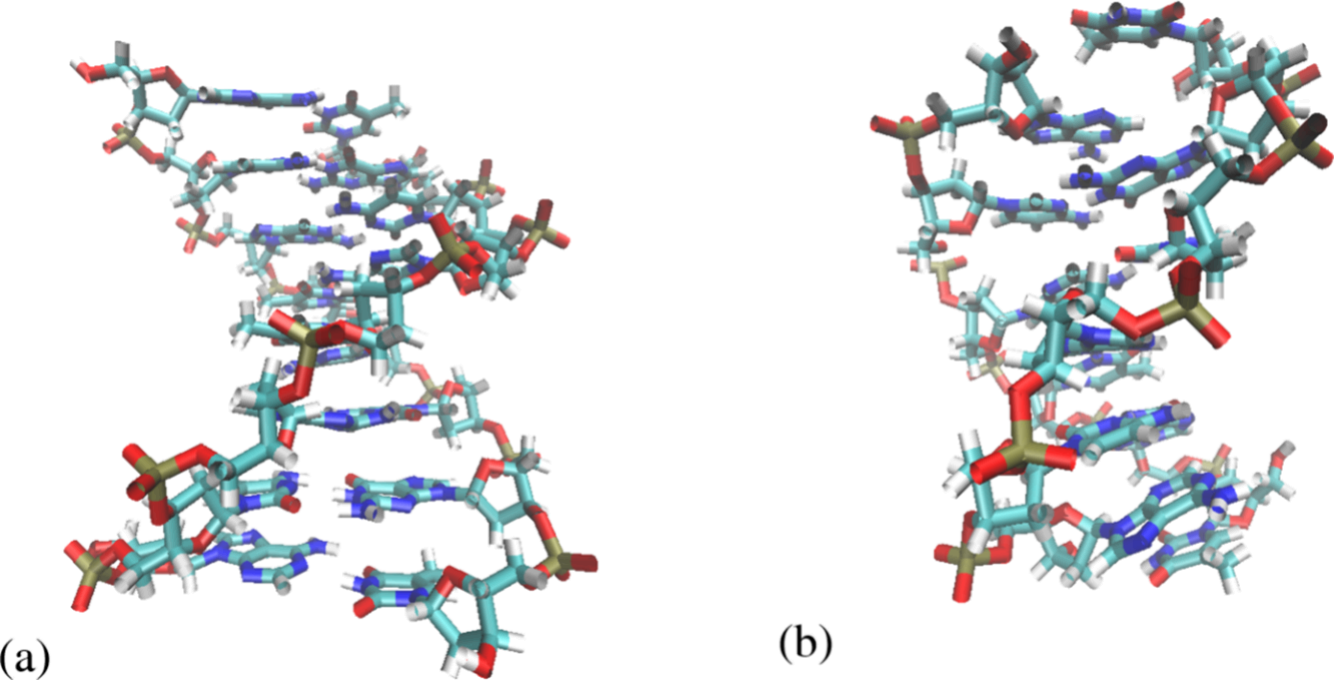
Full-atom visualization of the (a) 1AL9 and (b) 1AMD DNA oligonucleotides equilibrated in water.

The molecules simulated in the framework of this article are depicted in Figure 1. In addition to them, double-stranded oligonucleotide DNA sequences are considered herein, i.e., two structures downloaded from the Protein Data Bank under the code numbers 1AL9 [5′-d(AC|GTAC|GT)-3′, octamer DNA sequence]^37^ and 1AMD [5′-d(T|GT|ACA)-3′, hexamer DNA sequence]^37^ that have been also used to study interactions between anthracyclines and DNA.^37−40^ The symbol | indicates where the intercalation sites are located in the above-presented DNA sequences. A graphical representation of 1AL9 and 1AMD after equilibration is provided in Figure 2 using the VMD^41^ software with all the water molecules omitted for clarity. In order to help the reader better understand this article, all the systems described in this section are gathered in Table 1. Berubicin, as seen from Figure 1, is simulated in the neutral as well as in the protonated state. This was deemed necessary in order to make direct comparisons against results from a previous MD study of doxorubicin complexed with selected cyclodextrins.^15^ It is noted that at pH = 7.2, most of the berubicin (99.3%) is found in the protonated state, as obtained from the Henderson–Hasselbalch equation setting p*K*_a_ = 9.34.^42^ In general, pH values above 7 are representative of biological environments, such as blood (7.4),^7^ cell nucleus, and cytosol (7.2).^43^ The study of neutral berubicin in this article is mainly aimed at an investigation of the role of the drug’s charge in the mechanism of action. Secondarily, the comparison between the two charge states of the drug could give us leads in order to synthesize more effective anthracycline molecules. To this end, there are already experimental and computational studies that use both protonated and neutral anthracyclines.^44,45^ In the case of berubicin–cyclodextrin complexes in water, the constituent parts of the noncovalent complexes are initially placed apart (unbound state) and the spontaneous formation of the complex (bound state) is observed in the course of the MD simulations. This approach has been adopted for the formation of drug–cyclodextrin complexes via MD in other published articles in the literature.^15,35,46^ Two cyclodextrins widely employed in drug delivery are considered, i.e., 2-hydroxy-propyl-β*-*cyclodextrin (HP-β-CD) and γ-cyclodextrin (γ-CD); HP-β-CD is a broadly utilized derivative of β-cyclodextrin, while γ-CD presents a lipophilic interior cavity of larger diameter than that of HP-β-CD. As far as the complex formed between DNA and berubicin in an aqueous phase is concerned, the initial structures are created by utilizing the AutoDock program (version 4.2.6).^47^ The specific parameters chosen for the docking runs are provided in Section S1 of the Supporting Information of this article. It is also pointed out that the salt concentration (created by sodium and chloride ions) is set to 0.15 M in all the simulations concerning the berubicin–DNA complexes in the aqueous phase.

**Table 1:** Systems under Consideration in This Article

(1) protonated berubicin—DNA (1AL9, 1AMD)
(2) neutral berubicin—DNA (1AL9, 1AMD)
(3) protonated berubicin—HP-β-CD
(4) neutral berubicin—HP-β-CD
(5) protonated berubicin—γ-CD
(6) neutral berubicin—γ-CD

In all the systems under consideration herein, the water phase is described by the transferable intermolecular potential three-point (TIP3P) model,^48^ whereas the GLYCAM06^49^ and Amber parmbsc1^50^ force fields are used for the cyclodextrins and DNA oligonucleotides, respectively. Berubicin, either in the neutral or the protonated state, is simulated with the generalized Amber force field (GAFF).^51^ The antechamber and tLEaP modules of the Amber 14 package^52^ are employed for the creation of the topology files and the calculation of the atomic partial charges. Next, we make use of the ACPYPE script^53^ for the conversion of the above-mentioned topology files from Amber to GROMACS format. The drug–DNA systems contain 16331 water molecules, while the drug–cyclodextrin ones 5506 water molecules. As for the simulation boxes, they are constructed with the PACKMOL software.^54^ All the systems considered in this article are investigated *in silico* via full-atom MD simulations by making use of the GROMACS package 5.1.1.^55^ The production simulation for each system, whose duration is 1 μs, is carried out in the *NPT* statistical ensemble. Prior to the analysis of the MD trajectories, convergence checks are conducted, and both the results and the adopted methodology are presented in Sections S4 and S5 of the Supporting Information. As for the protocols and parameters of the equilibration and production runs, they are provided in Section S6.

As has already been mentioned above, special attention is paid to the thermodynamic description of the various complexes considered herein. The first method for the estimation of Gibbs energies is the LIE method,^56^ which is applied only for the drug–cyclodextrin complexes. In this method, the binding Gibbs energy, Δ*G*_bind_, is written as a linear combination of its van der Waals and electrostatic contributions:
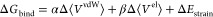
1

In order to calculate the time averages, ⟨*V*^vdW^⟩ and ⟨*V*^el^⟩, appearing in the above equation, two different MD simulations are required, i.e., one with the drug free in an aqueous phase and another one with the drug and receptor (cyclodextrin molecule) in the bound state in an aqueous phase. In more detail, Δ⟨*V*^vdW^⟩ stands for the difference between the averages of the Lennard-Jones interactions of the drug with its surroundings in each of the two aforementioned simulations, as it passes from its free aqueous state into the solvated complex. In the same manner Δ⟨*V*^el^⟩ is the difference between time averages of the electrostatic interactions. Δ*E*_strain_ is the cyclodextrin’s strain energy upon complexation. Technical details about its calculation are provided in ref (36). To extract it, the SANDER and cpptraj^57^ modules of Amber 14 are employed. The parameters α and β depend on the system under study, and the values employed in the context of this article are obtained from ref (36). The LIE method was also applied in the case of ref (15) giving very good agreement with published values for Δ*G*_bind_ in the case of doxorubicin–cyclodextrin complexes.

**Figure 3:**
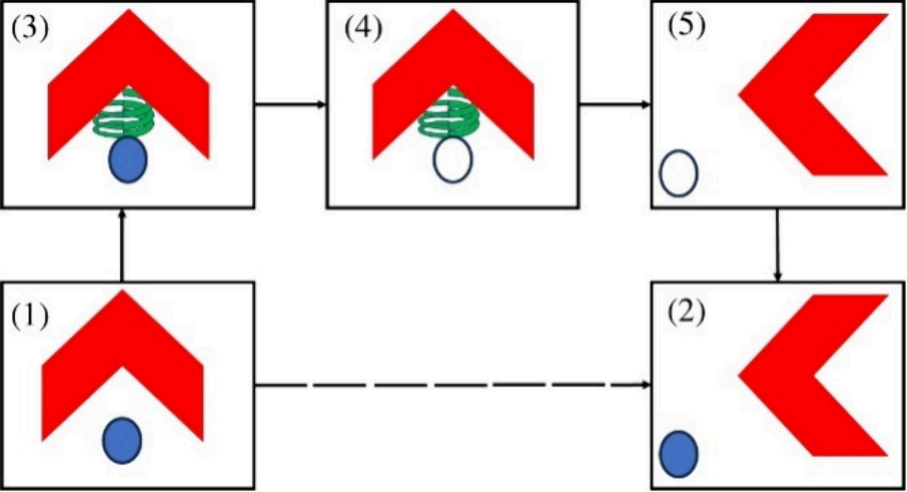
Thermodynamic cycle employed for the calculation of the binding Gibbs energies via DDM.^60^ The red and blue objects stand for the receptor and the ligand, respectively, with their interactions being completely turned on. The uncolored object with the blue contour is the ligand in which the nonbonded interactions are turned off. The green spring stands for the additional biasing harmonic potential between the molecules. The materialization of this thermodynamic cycle is executed via eq 2.

Another method for the calculation of Δ*G*_bind_ used in this article is DDM. This is based on the thermodynamic cycle sketched in Figure 3. A full description of the method in question may be found in refs (58−60), whereas useful technical details about its implementation in ligand–DNA complexes using the GROMACS package are given in ref (60). The binding Gibbs energy is written as a sum of four terms according to the intermediate steps of the thermodynamic cycle in Figure 3:
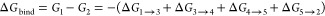
2Δ*G*_*i*→*j*_ = *G*_*j*_ – *G*_*i*_ is the transition Gibbs energy from state (*i*) to (*j*). The first term on the right-hand side of eq 2, Δ*G*_1→3_, is the Gibbs energy of switching on the harmonic distance restraint between berubicin and DNA in the bound state in water. Δ*G*_3→4_ stands for the free energy of decoupling the restrained drug from its surroundings by switching off all of its nonbonded Lennard-Jones and electrostatic interactions. This procedure is controlled by a coupling parameter, λ; λ = 0 indicates that the interactions are completely turned on, while when λ = 1, the interactions are fully turned off. Values of λ ∈ (0,1) correspond to gradually reduced interactions. The third term, Δ*G*_4→5_, concerns switching off the harmonic distance restraint while the berubicin’s interactions are still turned off, whereas the last term in eq 2, Δ*G*_5→2_ is the Gibbs energy of coupling the unbound drug to the solution. For charged ligands, such as the protonated berubicin, the binding Gibbs energy computed by eq 2 is modified according to the next equation:
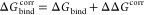
3ΔΔ*G*^corr^ accounts for the errors of electrostatic nature which arise from periodicity in the simulated solvent boxes.^60,61^ Technical details about the implementation of DDM in the systems under consideration herein are provided in Section S2 of the Supporting Information.

In addition to the previously reported thermodynamic methods, we make use of SBM for the estimation of the binding enthalpy, Δ*H*_bind_, as detailed in ref (62). In the framework of this method, the binding enthalpy is written in the following form:
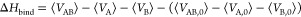
4where ⟨*V*⟩ indicates the average potential energy in each simulation which is described by the corresponding index. A and B stand for the receptor and the ligand of the complex, respectively, and AB stands for the complex itself; all of them are found in a water phase. When the indicator 0 is added to the index, the simulated system is the pure solvent with the same number of water molecules as in the case of A, B, and AB in water.

The thermodynamic analysis encompasses also the study of the desolvation effect that takes place when a drug is bound into a DNA sequence and more generally in the case of any complexation in a solvent environment. During this process, water molecules surrounding the drug and DNA are displaced to allow the formation of the complex, which has as a consequence the disruption of these water shells. The desolvation Gibbs energy, Δ*G*_des_, is written as follows:
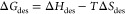
5where the desolvation enthalpy, Δ*H*_des_, and the desolvation entropy, Δ*S*_des_, are calculated by the next set of equations:
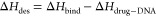
6a
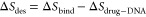
6bFor the calculation of Δ*H*_drug–DNA_, we take into account only the interactions between the drug and DNA, including the effect of volume change. The latter contribution, *P*Δ*v,* where *v* stands for volume, is considered negligible and consequently Δ*H*_drug–DNA_ ≃ Δ⟨*V*_drug–DNA_⟩. The entropy term Δ*S*_drug–DNA_, on the other hand, is the solutes’ configurational entropy difference from the drug and DNA in the unbound state to the formed complex. It is estimated by employing the weighted solvent accessible surface (WSAS) model, which considers the configurational entropy of a molecule as a weighted sum of the buried and nonburied solvent accessible surface area (SASA) of each atom. A full description of this method, as well as of the parameters in use, may be found elsewhere.^63^ A summary of this approach and its key-characteristics is provided in Section S3 of the Supporting Information.

## 3 Results and Discussion

The first part of this section begins with the results obtained from the molecular simulations of the protonated and neutral berubicin in DNA. As has already been reported, prior to MD, the initial configurations of the berubicin–DNA complexes are created by docking runs. Both the 1AMD and 1AL9 DNA sequences contain two intercalation sites. It is pointed out that in the latter sequence, the two intercalation sites are fully equivalent, while in the former one, there exists an intercalation site around the middle of the DNA sequence and another one toward the terminal regions. The docking runs show that when the drug is in these intercalation sites, the configurations are most stable from the thermodynamic point of view. Initially, the docking is blind; the whole DNA molecule is taken as the grid space. Next, we continue with specific-site docking in which each intercalation site is considered as the grid space; the drug explores the intercalation sites by sampling various conformational degrees of freedom so as to find the best configuration. The most stable docked configurations are depicted in Figure S1 of the Supporting Information. It is seen from the aforementioned figure that the anthraquinone group of berubicin is buried in the intercalation sites, whereas the rest of the molecule is located very close to the minor groove. The drug–DNA complex is also stabilized by the formation of a hydrogen bond network in the groove, as shall be described later in more detail in discussing the results from MD simulations. Such stabilization via hydrogen bonding is also observed in the case of other anthracycline molecules intercalated in DNA.^44,64−74^ The binding free energy estimates by the semiempirical free energy scoring function of AutoDock from the specific-site docking simulations are −17.6 and −17.8 kcal/mol for the two intercalation sites of the neutral berubicin–1AL9 complex and −18.2 and −18.3 kcal/mol for the same two intercalation sites of the protonated one. It is worth mentioning that the scores of the two intercalation sites are very similar in both the berubicin–1AL9 complexes due to their above-mentioned equivalency. The berubicin–1AMD complexes give a clear preference for the middle intercalation site, where the scores are −15.6 and −15.6 kcal/mol for the neutral and protonated berubicin cases, respectively, whereas the terminal intercalation sites’ binding free energies are −13.3 and −13.7 kcal/mol, respectively. As far as the input configurations for the molecular simulations are concerned, we choose those with the best docking scores; in the case of 1AL9, berubicin, in both charge states, is docked in one of the two equivalent interaction sites, whereas in the case of the 1AMD, the middle intercalation site is selected.

**Figure 4:**
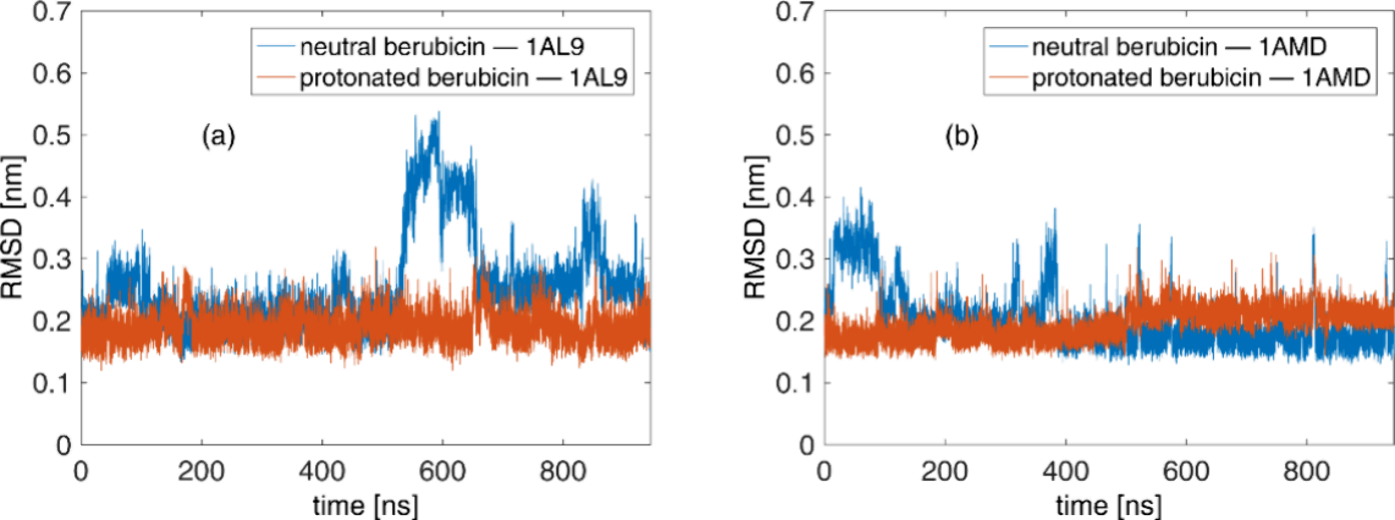
RMSD plots of the (a) berubicin–1AL9 complexes and (b) berubicin–1AMD ones.

**Figure 5:**
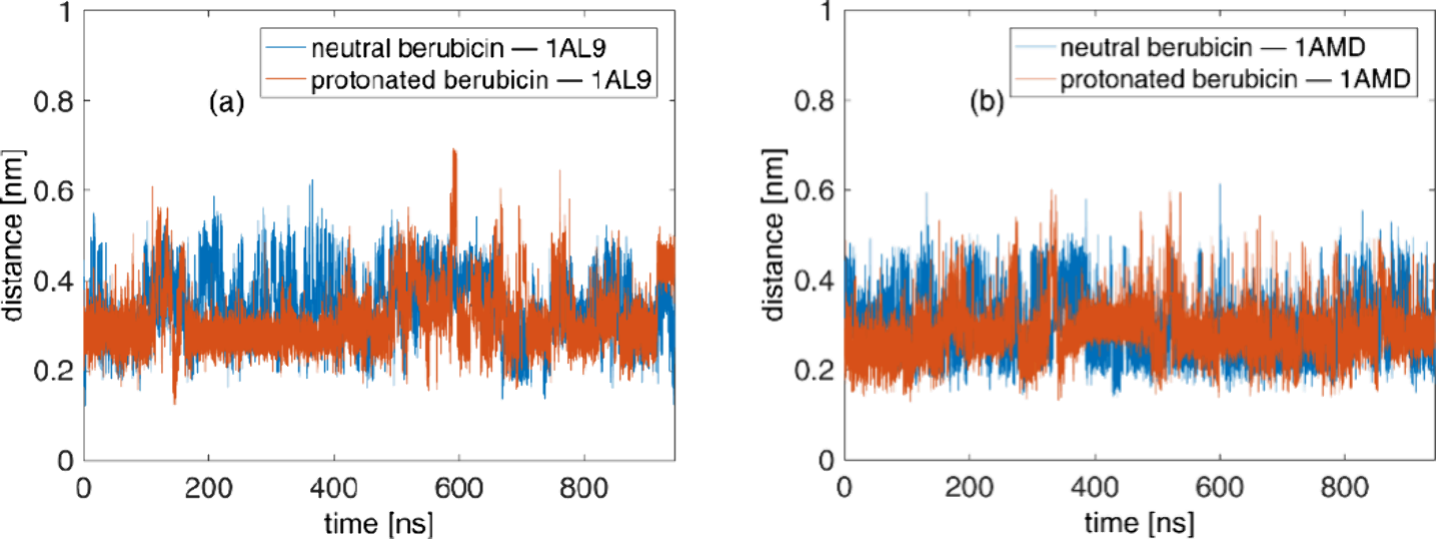
COM-to-COM distance between the intercalation site and berubicin: (a) berubicin–1AL9 and (b) berubicin–1AMD.

**Figure 6:**
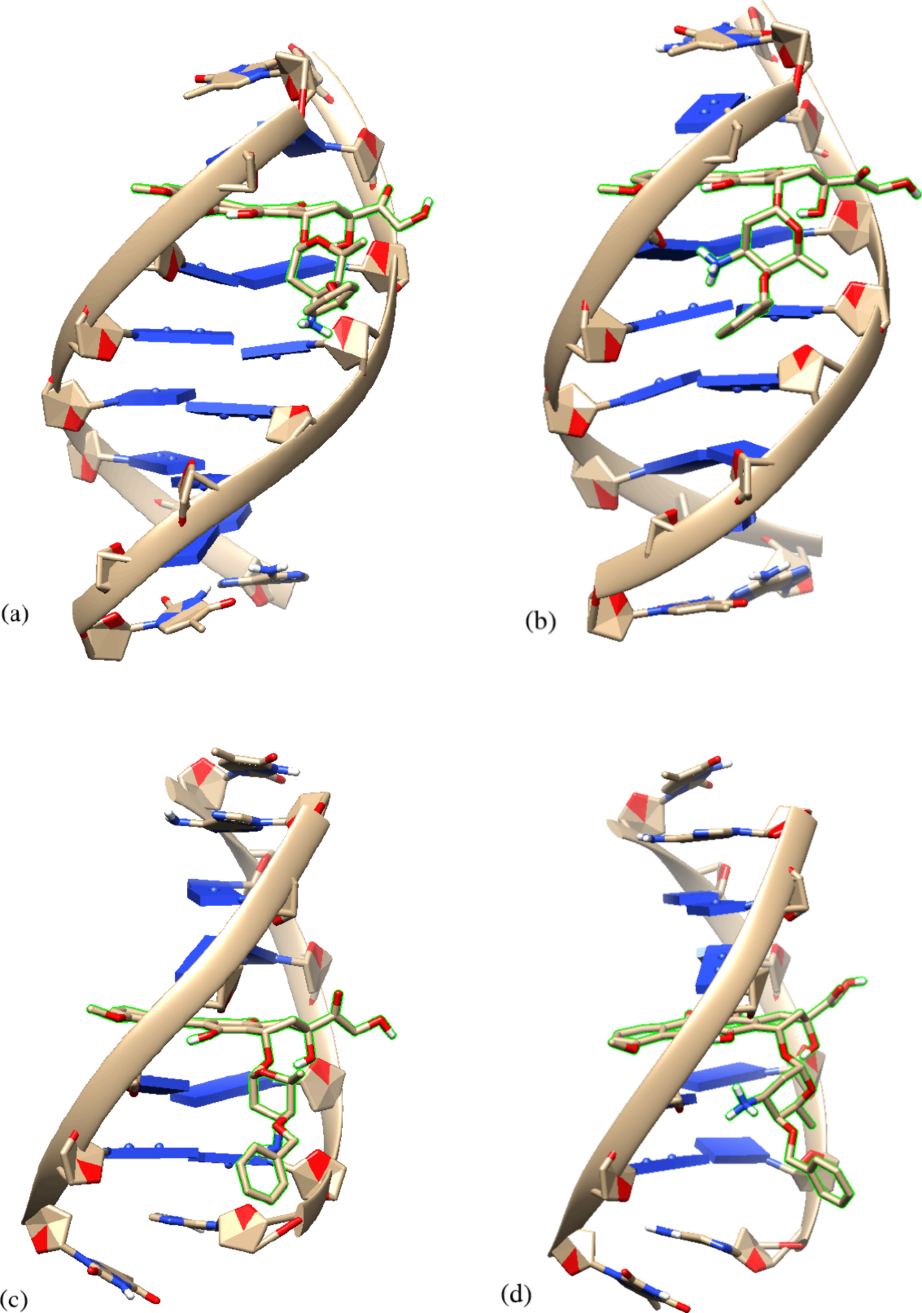
Graphical representations of berubicin bound in DNA as an intercalating agent obtained from a clustering analysis of the molecular simulations: (a) neutral berubicin–1AL9, (b) protonated berubicin–1AL9, (c) neutral berubicin–1AMD, and (d) protonated berubicin–1AMD.

Next, the analysis continues by studying the berubicin–DNA complexes, as sampled from the MD production runs in terms of selected structural and geometrical properties. To begin with, the root-mean-square deviation (RMSD) of all heavy atoms of the berubicin–DNA complexes, namely, all atoms except for the hydrogens, is calculated. RMSD plots of the two DNA sequences without berubicin are depicted as functions of the simulated time in the Supporting Information (see Figure S2), while those with berubicin intercalated in them are plotted in Figure 4. All the RMSDs are calculated with respect to the corresponding configurations obtained from the docking procedure. The nonzero values at the beginning of the production simulations (for time equals to 0) are due to the equilibration stage that always precedes them, as described in the previous section. As seen in Figures 4 and S2, all the RMSDs do not grow past the value of 0.6 nm throughout the simulation, indicating thus that the two DNA sequences are stable. To be more specific, RMSDs lie between 0.1 and 0.3 nm most of the simulation time, with neutral berubicin having a stronger effect on the RMSD of the DNA molecules studied herein. Another structural property investigated in the context of our analysis is the distance between the centers of mass (COMs) of the intercalation site and berubicin which is again recorded as a function of the simulated time and is shown in Figure 5. For all the complexes obtained from the equilibration stage, the COM-to-COM distance is fluctuating around 0.3 nm, indicating that berubicin remains well-buried in both the DNA sequences, regardless of its charge state. Representative configurations of the berubicin–DNA complexes are provided in Figure 6, as obtained from a clustering analysis conducted with GROMACS (“cluster” utility). As a next step, a study of the complexes is undertaken in terms of the characteristic groups of berubicin that are illustrated in Figure 1a. The hydrophobic anthraquinone group, which is a planar structure, is deeply buried in the intercalation site of the DNA sequences so as to avoid contact with the surrounding water molecules and develop stacking interactions with the complementary nitrogen-containing nucleobases. Another significant group of atoms is the anchor that is properly oriented in order to interact favorably with DNA, mainly through hydrogen bonds (further details are provided below). As for the sugar moiety, it is located in the minor groove of the DNA sequences, conferring further stability to the formed complex.

**Figure 7:**
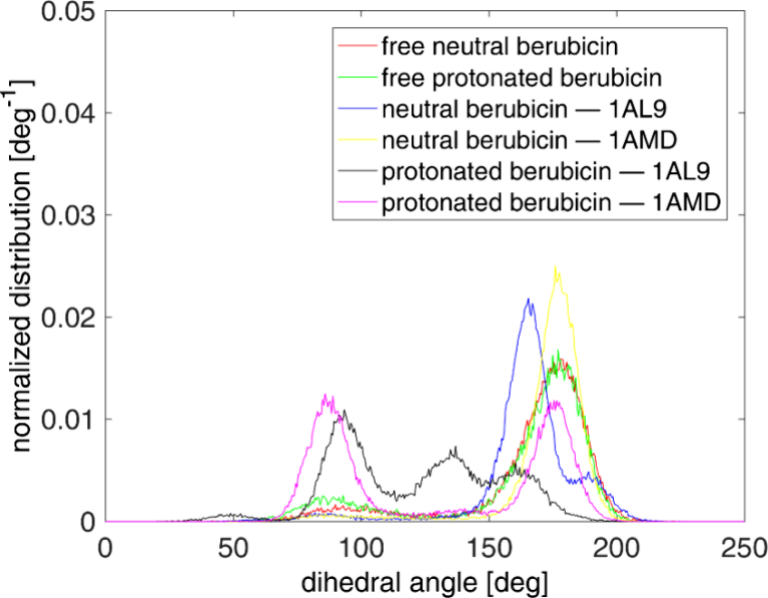
Normalized distribution of the dihedral angle describing the rotation around the glycosidic bond for the various berubicin–DNA complexes considered in this article. The dihedral angle of berubicin free in water is included as well.

**Figure 8:**
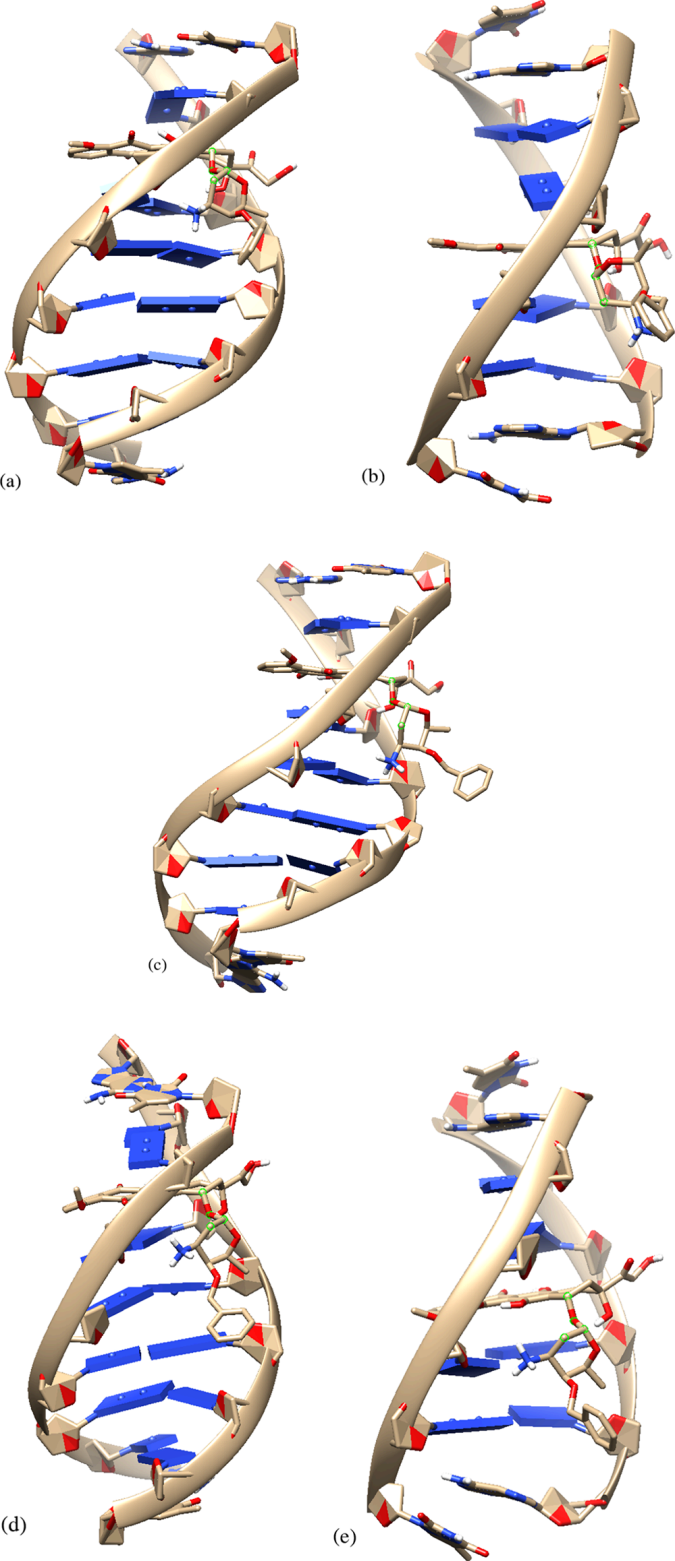
Representative configurations of the protonated berubicin–DNA complexes at the different stable conformational states. The glycosidic angle is highlighted with four green points, representing the involved atoms. (a) high glycosidic angle state (161°) of protonated berubicin–1AL9, (b) high glycosidic angle state (177°) of protonated berubicin–1AMD, (c) medium glycosidic angle state (137°) of protonated berubicin–1AL9, (d) low glycosidic angle state (93°) of protonated berubicin–1AL9, and (e) low glycosidic angle state (87°) of protonated berubicin–1AMD.

For a better understanding of the behavior of the sugar moiety and its groove-binding function, the rotation around the glycosidic bond, which exists in all the anthracyclines, is thoroughly investigated. To this end, we calculate the dihedral angle 1–2–3–4, which is annotated in Figure 1a, in the bound and unbound states. The significance of the glycosidic bond lies in the fact that its hydrolysis generates cardio-toxic products;^75^ in addition, the orientation of the anthracycline’s amino-group with respect to DNA determines the strength of the minor groove binding mode of the anthracycline.^72,75,76^ The normalized distributions of the dihedral angle being discussed are depicted in Figure 7, in which the trans conformation corresponds to 180°. When berubicin is free in water, the distributions exhibit a peak around 177°, as seen from the green and red curves of Figure 7. However, after the complexation, new distinct maxima emerge in the dihedral angle distributions of the berubicin–DNA complexes. As far as 1AMD complexes are concerned, protonated berubicin exhibits a new maximum around 90° and the relative populations corresponding to the two maxima are nearly equally distributed. In the case of 1AL9, protonated berubicin mostly adopts conformations around 90°, while it has significant populations at greater angles, too, with corresponding maxima. To clarify, we present representative configurations of all the conformers of the protonated berubicin in Figure 8, corresponding to the different stable conformational states from the maxima of Figure 7. Our analysis for the glycosidic bond is in agreement with ref (72) concerning daunorubicin in the protonated state, in which the tendency of the drug molecule to adopt significantly lower glycosidic dihedral angles upon intercalation, compared with its unbound state in water, is reported, which generates a new conformational state. In general, the results, shown herein agree with ref (77) on free daunorubicin and its analogues, in which the glycosidic dihedral angle possess high values in the range 162–168°. Additionally, the results reproduce the general tendency for the angle to take smaller values in comparison to its unbound state upon intercalation with DNA,^72,75,76^ in order for the sugar moiety to bend over the groove and increase its contacts with it. Finally, regarding the neutral berubicin–DNA complexes, the rotation around the glycosidic bond is of similar shape to that of unbound berubicin in the water phase, due to the weaker interactions of the neutral drug with the minor groove of the DNA sequences.

It is known from the literature that, in general, anthracyclines form a strong hydrogen bond network when bound to DNA.^44,64−74^ In this article, for the formation of a hydrogen bond, standard geometrical criteria are applied by making use of the “hbond” tool of GROMACS. More precisely, these criteria are the distance between the donor and acceptor being less than 0.35 nm and the angle among donor, hydrogen, and acceptor being less than 30°. In the analysis detailed in this section, the abbreviation HB stands for the average number of hydrogen bonds and the subscript in HB points out which pair of molecules or group of atoms is referred to. Various average numbers of hydrogen bonds are gathered in Table 2 concerning the hydrogen bond network formed by berubicin with both DNA and water in the bound and unbound states. The anchor and sugar moieties of berubicin play the most significant role in the hydrogen bonding and nearly 75% of the hydrogen bonds with the water molecules in the DNA-free aqueous phase originate from these two groups. It is also noteworthy that there exist approximately 1.7 intramolecular hydrogen bonds in the anthraquinone group formed between the carbonyl and hydroxyl groups (see also Figure 1a) of the protonated and neutral berubicin. This is verified by other published articles^73,78,79^ regarding anthracyclines, where it is reported that these intramolecular hydrogen bonds contribute to the stability of the anthracycline–DNA complexes because they act protectively by hindering the entry of water molecules in the intercalation site. In the bound state, a decrease by 25–35% is observed in the number of hydrogen bonds between berubicin and water due to steric effects caused by the insertion of the drug in the intercalation site of DNA. Simultaneously, the percentage of hydrogen bonds with water formed by the anchor and sugar moieties over the total hydrogen bonds of berubicin with water increases from 75% to nearly 85–90%; this is attributed to the more effective coverage of the anthraquinone group into the intercalation complex compared with the anchor and sugar moiety ones. Another observation from the molecular simulations is the water-uptake effect, i.e., the attraction of water molecules to the neighborhood of the drug–DNA complex. This effect has been reported and discussed in a number of experimental and computational studies concerning anthracyclines,^80−82^ and for that reason, it is also investigated in this article. Ref (80), in particular, analyzes the water-uptake effect in terms of the hydrogen bond difference between the intercalation site and water in the unbound and bound states of the complex. In our analysis, 5′-d(AC|G)-3′ in 1AL9 and 5′-d(T|AC)-3′ in 1AMD base-pair sequences are taken into account; for all the complexes under consideration herein, the increase in the number of hydrogen bonds is about 4–5, while, according to ref (80), the corresponding increase in the case of DNA–daunorubicin complexes is found to be about 8.

**Table 2:** Average Numbers of Hydrogen Bonds Formed in the Berubicin–DNA Complexes^a^^,^^b^

	HB_anthraq–wat_	HB_anthraq–DNA_	HB_(anch+sug)–wat_	HB_(anch+sug)–DNA_
unbound neutral berubicin	2.62 ± 0.03		7.32 ± 0.02	
unbound protonated berubicin	2.51 ± 0.01		8.03 ± 0.07	
neutral berubicin–1AL9	1.15 ± 0.24	<0.1	5.31 ± 0.14	1.66 ± 0.10
protonated berubicin–1AL9	1.01 ± 0.14	<0.1	6.66 ± 0.11	1.69 ± 0.09
neutral berubicin–1AMD	0.98 ± 0.23	<0.1	5.79 ± 0.13	0.78 ± 0.03
protonated berubicin–1AMD	0.52 ± 0.06	<0.1	6.71 ± 0.04	0.67 ± 0.06

a For comparison purposes, hydrogen bonds of berubicin in the unbound state are given in the first two rows.

b The abbreviations anthraq, anch, and sug refer to the anthraquinone, anchor, and sugar moieties of berubicin, whereas wat stands for water. The symbol HB_*x*–*y*_ stands for the hydrogen bonds between *x* and *y*.

The thermodynamic description of the berubicin–DNA complexes begins with the calculation of the binding Gibbs energy according to eqs 2 and 3 (in the framework of DDM); the Δ*G*_bind_ values are gathered in Table 3 along with their enthalpic and entropic contributions. It is seen from this table that, in the case of protonated berubicin, the binding Gibbs energies are very close and within the statistical error for both 1AL9 and 1AMD sequences (Δ*G*_bind_ ≃ −65 kJ/mol). Δ*G*_bind_ is −20.1 kJ/mol for the neutral berubicin–1AL9 complex, indicating that the interactions between the constituent parts of the DNA complex are significantly less favorable compared with protonated berubicin. DNA, as a polyanion, forms a more stable noncovalent complex when the positively charged berubicin is intercalated in it. One of the advantages of DDM is that the free energy terms Δ*G*_3→4_ and Δ*G*_5→2_ are further decomposed into contributions from the van der Waals and electrostatic interactions, namely, Δ*G* = Δ*G*_vdW_ + Δ*G*_el_ (see Section S2 of the Supporting Information for more details). In the Supporting Information, Δ*G*_vdW_ and Δ*G*_el_ of the aforementioned Gibbs energies are presented (Table S1). The dominant term, even in the two complexes of protonated berubicin, is the van der Waals contribution, thus indicating that the principal driving force for the complexation is hydrophobic interactions, as expected by the transfer of a hydrophobic molecule into a hydrophobic binding pocket. The nonpolar interactions in the anthracycline–DNA intercalation complexes, such as direct van der Waals forces and the hydrophobic effect, are also mentioned in many studies as essential driving forces of intercalation^45,65,74,83−89^ and the most determining ones especially,^45,65,74,83,85−87,89^ even when the anthracycline intercalator is positively charged.^45,65,74,83,87^ However, secondarily, the electrostatic interactions in the case of protonated berubicin are also included in the driving forces of the complexation, as is also mentioned in a plethora of studies and especially on protonated anthracyclines with DNA.^45,65,66,73,83,85−88^ Another finding from the thermodynamic analysis is that the binding Gibbs energies are similar for the two DNA sequences (within statistical error). This can be compared with refs (90−92), in which doxorubicin and daunorubicin, both of them belonging in the class of anthracyclines, exhibit close binding Gibbs energies with the poly deoxyribonucleotides poly[d(GC)] and poly[d(AT)] with the difference between these two being 1–2 kJ/mol for daunorubicin and doxorubicin, because 1AL9 contains the d(C|G) intercalation site and 1AMD contains the d(T|A) one. It is worth mentioning, however, that more than just two base-pairs contribute to the intercalation Gibbs energy.^87,90,92−94^ To the best of our knowledge, there do not exist experimental studies reporting binding Gibbs energies for the berubicin–DNA complexes. Concerning doxorubicin and daunorubicin, there is a rich literature concerning binding Gibbs energies in double-stranded DNA sequences. As far as doxorubicin is concerned, experimental and computational Δ*G*_bind_ fall in the ranges from −36.4 to −36.7 kJ/mol^44,90^ (the experimental conditions are salt concentration of 0.1–0.15 M, pH of 7.0–7.4, and temperature of 293–298 K) and from −26 to −59 kJ/mol^38,45,65,87,93^ (the computational conditions are similar, with a salt concentration of 0.1–0.2 M, a temperature 297–310 K, and a pressure of 1 bar), respectively. In the case of daunorubicin, the published experimental values are in the range from −24 to −45 kJ/mol^44,66,91,92,95−98^ (the experimental conditions are a salt concentration of 0.15–0.20 M, a pH of 7, and a temperature of 293–298 K), whereas the *in silico* estimated binding Gibbs energies take values from −34 to −49 kJ/mol^38,80,83,93^ (the conditions are a salt concentration of 0.15 M, a temperature of 297–300 K, and a pressure of 1 bar). The more negative Δ*G*_bind_ values of berubicin with respect to those of doxorubicin and daunorubicin are interpreted based on the higher hydrophobicity (predicted octanol/water partition coefficient of berubicin is 2.64, while this is 1.41 and 1.68 for doxorubicin and daunorubicin, respectively)^42^ as follows. The van der Waals interactions of the complexes formed between berubicin and DNA are stronger due to the higher hydrophobicity of berubicin over the two above-mentioned anthracyclines, as well as the smaller desolvation Gibbs energy of berubicin, as discussed in more detail below in this section. Another reason for the stronger binding of berubicin is that a larger portion of this molecule, compared to those of doxorubicin and daunorubicin, is located inside the minor groove of the DNA sequences, due to the additional substitution of berubicin (see Figure 1a).

**Table 3:** Binding Gibbs Energy, Enthalpy, and Entropy for the Berubicin–DNA Complexes under Consideration in This Article

system	Δ*G*_bind_ [kJ/mol]	Δ*H*_bind_ [kJ/mol]	Δ*S*_bind_ [J/mol/K]
neutral berubicin–1AL9	–20.1 ± 3.1	–53.4 ± 24.1	–107.4 ± 78.4
protonated berubicin–1AL9	–63.2 ± 4.5	–98.3 ± 19.6	–113.2 ± 64.9
protonated berubicin–1AMD	–65.7 ± 5.3	–88.5 ± 13.6	–73.5 ± 47.1

As mentioned in Section 2, the binding enthalpy may be estimated by employing SBM. The combination of SBM and DDM allows the calculation of the binding entropy through the equation: . From the binding enthalpies and entropies gathered in Table 3, it is derived that the intercalation of the protonated berubicin is mainly enthalpy-driven, which is in accordance with other anthracyclines intercalated to DNA.^80,91,99,100^ Generally, it holds that Δ*H*_bind_ ≪ 0 and Δ*S*_bind_ < 0 in intercalations.^101,102^ Furthermore, it is extracted that Δ*H*_bind_/Δ*G*_bind_ ≃ 1.3–1.6 for protonated berubicin in 1AL9 and 1AMD. In ref (102), the above-mentioned ratio lies in the range 0.83–1.97 for a number of intercalators in which both doxorubicin and daunorubicin are included. Concerning neutral berubicin, again the binding entropy is negative but the ratio Δ*H*_bind_/Δ*G*_bind_ ≃ 2.7 is greater than those of the protonated berubicin.

From the thermodynamic point of view, the desolvation effect contributes positive Gibbs energies and acts as a barrier in the complexation process. This is expected from the highly unfavored term of desolvation in continuous-solvent approaches extracting Gibbs energy, such as the Molecular-Mechanics Poisson–Boltzmann Surface-Area (MM-PBSA) method or the Molecular-Mechanics Generalized-Born Surface-Area (MM-GBSA) method in computational studies (some examples implementing these methods in anthracycline–DNA intercalation complexes are found in refs (65,74,83,87,88), and all of them report positive desolvation Gibbs energies). This effect is more pronounced when the drug is protonated since the charged molecules prefer being in the aqueous phase to a greater extent than the corresponding neutral ones. The desolvation Gibbs energies, enthalpies, and entropies for all the complexes, as estimated by eqs 5, 6a and 6b, are given in Table 4. First, it is observed that all the entries in this table are positive and Δ*G*_des_ is significantly higher in the protonated berubicin cases for the reason that the solvation shell is more difficult to break. The desolvation entropies are positive due to the hydrophobic effect; when a hydrophobic molecule leaves the aqueous phase, the entropy of the system increases, since the water molecules of the solvation shell are entropically trapped.^103^ As for the desolvation enthalpies, they are also positive because the interactions between berubicin and water are significantly hindered upon intercalation. Qualitatively, these results are in agreement with the literature^104−107^ regarding the desolvation thermodynamics in complexations. In particular, positive entropies due to the hydrophobic effect^84,89^ and mainly positive enthalpies (in some cases due to the strong hydrophilic interactions between the solutes and water) have been reported.

**Table 4:** Desolvation Thermodynamic Properties

system	Δ*H*_des_ [kJ/mol]	Δ*S*_des_ [J/mol/K]	Δ*G*_des_ [kJ/mol]
neutral berubicin–1AL9	+164.0 ± 28.5	+17.6 ± 79.0	+158.5 ± 37.6
protonated berubicin–1AL9	+268.3 ± 29.7	+39.9 ± 65.8	+255.9 ± 32.2
protonated berubicin–1AMD	+263.9 ± 19.0	+67.2 ± 47.7	+243.1 ± 24.1

**Figure 9:**
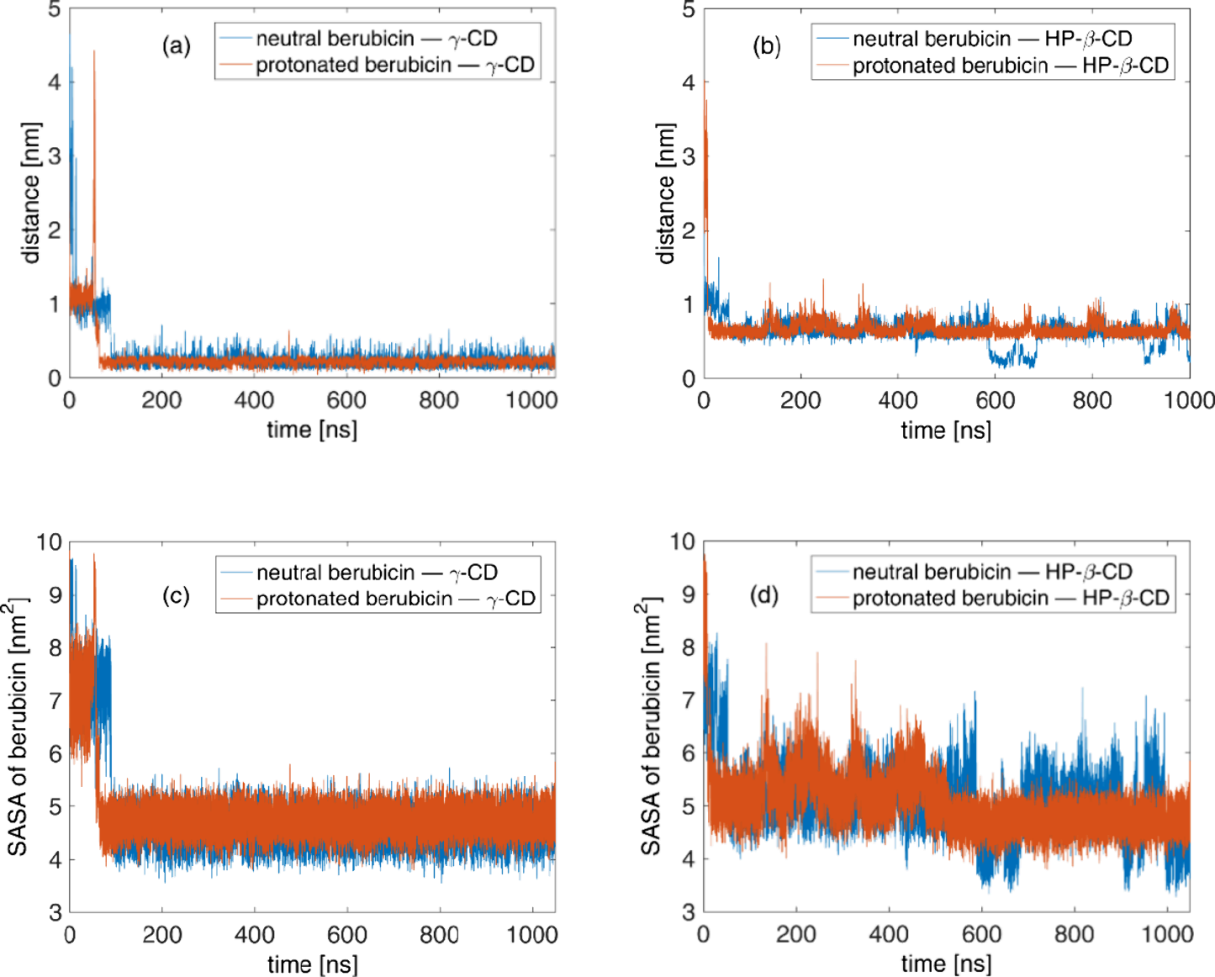
Time evolution of the complex formation indicators. (a), (b) COM-to-COM distance of the berubicin and cyclodextrin molecules. (c), (d) SASA of berubicin.

**Figure 10:**
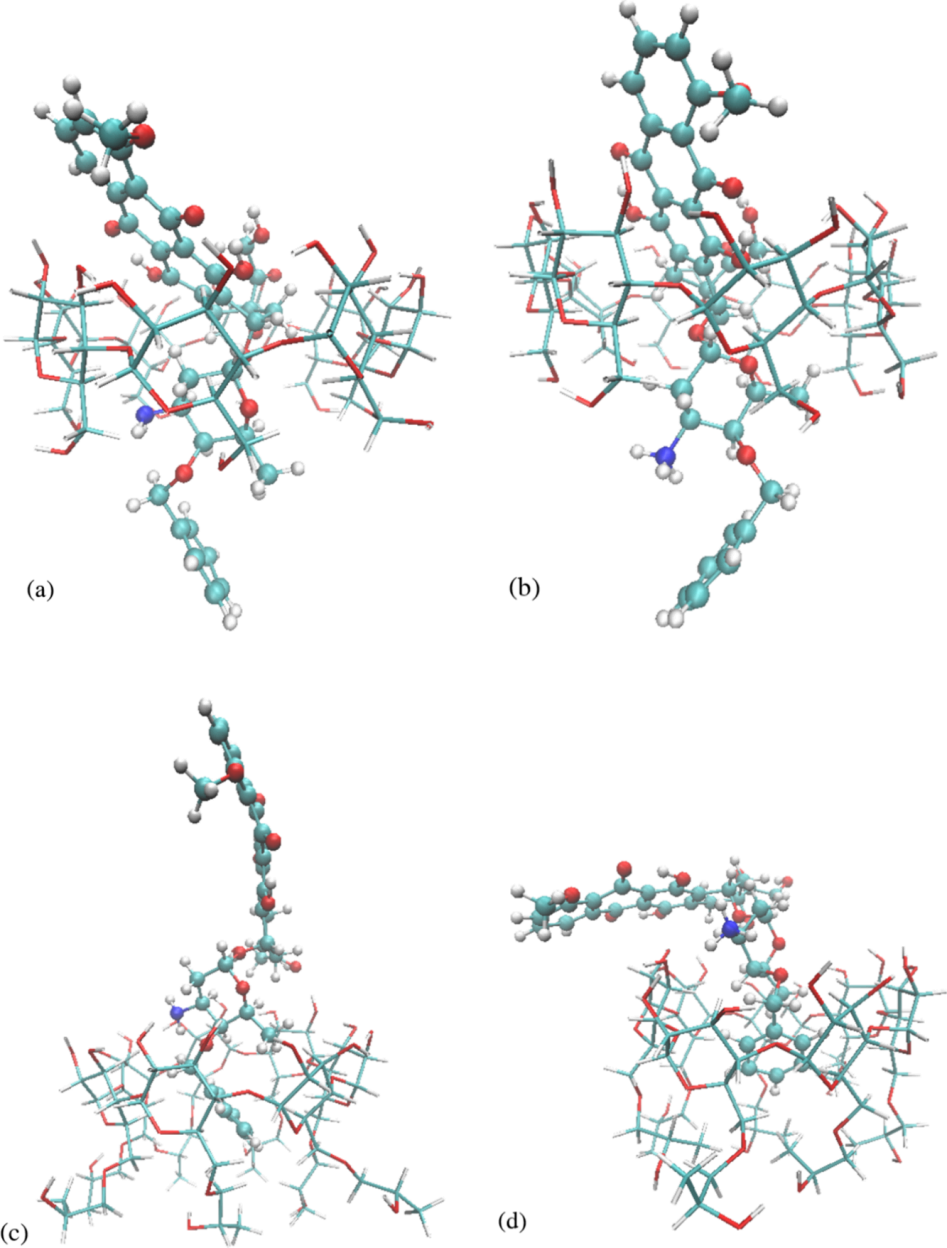
Representative configurations of the following complexes: (a) neutral berubicin−γ-CD, (b) protonated berubicin−γ-CD, (c) neutral berubicin–HP-β-CD, and (d) protonated berubicin–HP-β-CD.

As mentioned above, the drug being discussed is also simulated with selected cyclodextrins in an aqueous phase. The analysis of the results begins with the complexation process itself by monitoring, first, the COM-to-COM distance between berubicin and cyclodextrin molecules, as a function of the simulation time; the reader is reminded that in all considered cases the constituents of the berubicin–cyclodextrin complexes are initially placed in water in the unbound state. The COM-to-COM distance in the course of the MD simulations is plotted in Figure 9a,b, concerning γ-CD and HP-β-CD, respectively, as host molecules. At the beginning, the neutral as well as the protonated berubicin molecules are free in the water phase. After a few nanoseconds, the drug enters spontaneously into the hydrophobic cavities of the cyclodextrins to form supramolecular complexes. It is pointed out that, after the formation of the complexes, no dissociations are recorded during the simulations. In the special case of HP-β-CD, the position of the center of mass is computed, not taking into account the atoms of the substitutes (see also Figure 1c). It is extracted that in the bound state, the time average of the COM-to-COM distance is equal to 0.215 ± 0.002, 0.219 ± 0.003, 0.640 ± 0.005, and 0.657 ± 0.010 nm for the neutral berubicin−γ-CD, protonated berubicin−γ-CD, neutral berubicin–HP-β-CD, and protonated berubicin–HP-β-CD complexes, respectively. From these distances, it is seen that in γ-CD complexes, the drug dives much more deeply into the hydrophobic cavity. Representative configurations, as obtained from a “cluster” analysis with the “cluster” tool of GROMACS over all frames in the bound state, are seen in Figure 10. The findings from Figure 10 are further discussed below in terms of other properties.

For a more detailed understanding of the complexation process with cyclodextrins, in addition to the COM-to-COM distance, the SASA of berubicin in the neutral and protonated states is monitored in the course of the MD simulations by employing the *sasa* tool^108,109^ of GROMACS. The results are presented in Figure 9c,d for the berubicin−γ-CD and berubicin–HP-β-CD complexes, respectively. In both cases, a drop in the SASA values is observed due to the inclusion of the drug into the cavity, which makes its surface less accessible to water molecules. In the unbound state, the SASA of berubicin is approximately 9 nm^2^, which corresponds to the SASA of free berubicin in water. Upon complexation, the SASA average values of berubicin decrease to 4.570 ± 0.007, 4.722 ± 0.008, 5.155 ± 0.020, and 5.259 ± 0.049 nm^2^ for the neutral berubicin−γ-CD, protonated berubicin−γ-CD, neutral berubicin–HP-β-CD, and protonated berubicin–HP-β-CD complexes, respectively. Berubicin−γ-CD complexes exhibit lower values than the berubicin–HP-β-CD ones because berubicin enters more deeply into the cavity, and therefore, a smaller area is accessible to the surrounding water phase in the former case. The SASA analysis is further extended by taking into account also the cyclodextrin molecules. For validation purposes, the SASA values of γ-CD and HP-β-CD free in water were calculated. The results are presented in Table 5, in which corresponding published values from other studies are given in parentheses for comparison purposes.^110,111^ In Table 5, the SASA values of the supramolecular complexes, as a whole, are reported as well; a conclusion drawn from these values is that berubicin becomes more soluble due to the significant increase in SASA value as it goes from the free to the complex state. This trend is also reported in published articles concerning other drug–cyclodextrin complexes.^15,35,112^ A measure of the effectiveness of the drug inclusion is the SASA change, Δ*S*ASA, defined as follows:^110^^,^^111^
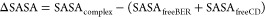
7The Δ*S*ASA average values are −8.292 ± 0.014, – 7.967 ± 0.20, – 5.807 ± 0.207, and −5.906 ± 0.212 nm^2^ for the neutral berubicin−γ-CD, protonated berubicin−γ-CD, neutral berubicin–HP-β-CD, and protonated berubicin–HP-β-CD complexes, respectively. It is noted that this quantity is negative in all the complexes, as anticipated. The reason is the existence of atomic contacts between the constituent molecules of the complexes since solvent molecules are not able to penetrate easily between contacted atoms. The negativity of this quantity is discussed in other *in silico* studies too and it is known to be associated with the contact surface of the molecules, which is directly related with the hydrophobic interactions.^111,113,114^ It is known that doxorubicin derivatives undergo dimerization,^32^ which has as a consequence the reduction of their anticancer action.^115^ A proposed mechanism for the anthracycline dimerization involves a parallel or antiparallel orientation of the anthraquinone groups of the two molecules.^32^ According to the literature, a cyclodextrin is able to prevent this kind of dimerization by including aromatic groups in its cavity and therefore hindering the π–π interactions between the aromatic compounds.^116^ Also, the glycosidic bond (see Figure 1a) of anthracyclines decreases their stability because it undergoes glycosidic degradation in water.^28^ Thus, in order to check whether cyclodextrins act protectively toward berubicin, the SASA changes are calculated separately for the anthraquinone group and the glycosidic oxygen of berubicin, which are marked in Figure 1a. The anthraquinone group is found to be buried by (39.0 ± 0.3), (45.1 ± 0.3), (14.7 ± 0.9), and (18.8 ± 1.2)% for the neutral berubicin−γ-CD, protonared berubicin−γ-CD, neutral berubicin–HP-β-CD, and protonated berubicin–HP-β-CD complexes, respectively, while the corresponding SASA burials for the glycosidic oxygen are (99.7 ± 0.1), (98.7 ± 0.2), (61.4 ± 4.5), and (82.4 ± 5.9)%, respectively. In all these cases, the degree of inclusion of the aforementioned groups is satisfactory enough to prevent or reduce the undesired effects of berubicin as an anthracycline drug.

**Table 5:** SASA Values of Berubicin and Cyclodextrins in the Bound and the Unbound States^a^

(supra)molecule	SASA (nm^2^)
free neutral berubicin	9.043 ± 0.002
free protonated berubicin	9.090 ± 0.002
free γ-CD	13.657 ± 0.012 (12.68)^111^
free HP-β-CD	15.115 ± 0.151 (16.04)^110^
neutral berubicin−γ-CD complex	14.410 ± 0.011
protonated berubicin−γ-CD complex	14.783 ± 0.018
neutral berubicin–HP-β-CD complex	18.293 ± 0.055
protonated berubicin–HP-β-CD complex	18.194 ± 0.071

a The values reported in parentheses are obtained from other published studies.

There is a plethora of computational and experimental studies concerning cyclodextrin complexation with doxorubicin. It has been found that the complexation proceeds in such a way that the anthraquinone group or a part of it is inserted into the cavity.^15,30,117−120^ This is confirmed here for berubicin via both the SASA analysis and Figure 10. Cyclodextrin complexation has been found to reduce the above-mentioned undesirable aspects of doxorubicin and doxorubicin-derivatives;^25,30,120−122^ it enhances water solubility and stability in aqueous environments and prevents the aforementioned dimerization. The above observations clearly suggest that the hydrophobic groups of berubicin are included into the hydrophobic cavities of cyclodextrins, whereas the hydrophilic groups lie in the surrounding water phase (see Figure 10). This indicates that hydrophobic interactions constitute main driving forces for this kind of supramolecular complexation, which is bibliographically verified for the cyclodextrin complexations.^21,123^ The main difference between the two charge states of berubicin is that its amino-group is covered more efficiently in the neutral complex by γ-CD, while it is more exposed to water in the protonated state; this difference is visualized in Figure S3. Due to that, the average SASA of the amino-group decreases by (72 ± 2) and (35 ± 2)% in the neutral berubicin−γ-CD and the protonated berubicin−γ-CD complexes, while the COM-to-COM distances between the amino-group and γ-CD are (0.434 ± 0.009) and (0.604 ± 0.035) nm, respectively. Therefore, the amino-group is located further from the cavity in the protonated case, resulting in stronger interactions with water, as also discussed below in the hydrogen bond analysis. The same finding is reported in ref (35) regarding the fluoxetine−β-cyclodextrin complex in both the neutral and protonated charges of fluoxetine.

**Figure 11:**
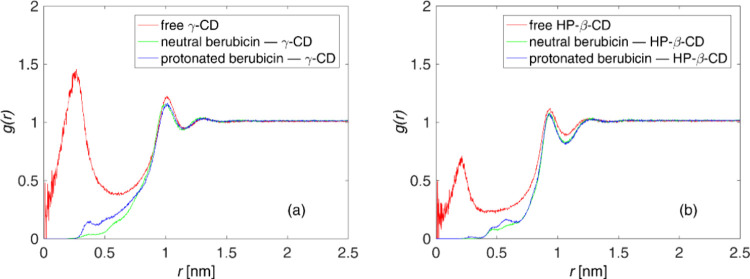
RDF of water molecules around the COM of (a) γ-CD and (b) HP-β-CD in their unbound and bound states in water. For HP-β-CD cases, the substituents’ atoms are not counted into the COM.

Part of the complexation process is the dislocation of entrapped water molecules from the interior of the lipophilic cavity of cyclodextrins. This has been observed in molecular simulations of doxorubicin–cyclodextrin,^15^ as well as fluoxetine–cyclodextrin^35^ complexes in water solutions. Furthermore, this finding is bibliographically confirmed.^21,123^ The analysis presented in this paragraph is based on radial distribution functions (RDFs), *g*(*r*), of the pair: water oxygen and COM of the selected cyclodextrin (either γ-CD or HP-β-CD, neglecting the substituents again). The number of entrapped water molecules, ⟨*N*_wat_⟩, is calculated in terms of RDF by using the equation:
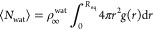
8The parameter *R*_eq_ in the definite integral stands for the equivalent radius of the cyclodextrin, calculated as the half of each cyclodextrin’s internal diameter,^124^ i.e.,: 0.484 ± 0.001 nm (γ-CD) and 0.399 ± 0.002 nm (HP-β-CD). ρ_∞_^wat^ is the bulk number density of water oxygen atoms. Figure 11 depicts the RDF, *g*(*r*), in the unbound and bound states for both cyclodextrins taken into account. The first peaks at small distances (*r* < 0.5 nm) in the free cyclodextrin cases are due to the entrapped water; they vanish in the formed complexes, due to the water dislocation. However, at higher distances, the plotted RDFs in Figure 11 remain unchanged; this means that the external water shell is not disturbed upon complexation. To quantify this effect, the number of the entrapped water molecules, ⟨*N*_wat_⟩, is calculated for the unbound and bound states according to eq 8. ⟨*N*_wat_⟩ is found equal to 11.9 ± 0.1 and 3.0 ± 0.1 for guest-free γ-CD and HP-β-CD, respectively; these values agree with published *in silico* studies.^125−127^ In the bound state, the number of entrapped water molecules drops to 0.4 ± 0.1, 1.4 ± 0.1, and <0.1 for the neutral berubicin−γ-CD, protonated berubicin−γ-CD, and both the berubicin–HP-β-CD complexes. It is inferred from the above analysis that the berubicin−γ-CD complexes contain more cavity waters than those of HP-β-CD due to the bigger cavity of γ-CD.

Another aspect of the complexation process is the hydrogen bonding. The analysis presented in this paragraph is similar to that adopted in refs (15,35) concerning drug–cyclodextrin complexes. The results are presented in Table 6, in which various hydrogen bond pairs in both the unbound and bound states of the molecules are listed. For validation purposes, we initially calculate the hydrogen bonds formed by γ-CD and HP-β-CD with water as 36.45 and 36.04, respectively; these compare well with published data,^125,128,129^ as shown in Table 6. In the unbound state, berubicin forms approximately 10 hydrogen bonds with water. Upon complexation in γ-CD, the aforementioned number of hydrogen bonds drops to 5.22 and 6.71 for the neutral and protonated forms of berubicin, respectively. This is explained as follows: the drug, when entering the cavity of cyclodextrin, is less accessible to the surrounding water molecules, while the displacement of entrapped water, as discussed above in this section, does not allow such hydrogen bonds inside the cavity. A drop is also observed in the number of hydrogen bonds formed between the selected cyclodextrins and water after the complexation, and this is common in cyclodextrin complexations in which the ligand is able to disturb the hydrogen bond network of the cyclodextrins.^15,35,130^ In addition to the displacement of entrapped water, the segments of berubicin that fall outside the cavity cause a weaker hydrogen bond network between the cyclodextrin and water molecules. The aforementioned trends in hydrogen bonding are observed as well in the case of doxorubicin with the same cyclodextrins, as reported in a recent *in silico* study.^15^ In ref (131), it is reported that a significant interaction in the doxorubicin−γ-CD complex stems from the intermolecular hydrogen bonds between the aglycone of doxorubicin and the hydroxyl groups of γ-CD (see also Figure 1). Herein, this is verified as well in the case of berubicin−γ-CD complexes as well since these hydrogen bonds contribute (55 ± 1)% (neutral berubicin) and (60 ± 2)% (protonated berubicin) of the total intermolecular hydrogen bonds of the complexed molecules. Another conclusion drawn from Table 6 is that the berubicin−γ-CD complex is stronger than berubicin–HP-β-CD in terms of hydrogen bonding.

**Table 6:** Average Numbers of Hydrogen Bonds Formed in the Various Berubicin–Cyclodextrin Systems^a^^,^^b^

molecule (mol)	HB_mol–wat_	HB_ber–CD_
neutral berubicin (unbound)	9.94 ± 0.02	
protonated berubicin (unbound)	10.54 ± 0.07	
γ-CD (unbound)	36.45 ± 0.09 (36–37)^125,128^	
HP-β-CD (unbound)	36.04 ± 0.15 (35.2)^129^	
neutral berubicin (bound)	5.22 ± 0.06	3.04 ± 0.06
γ-CD (bound)	28.76 ± 0.08	
protonated berubicin (bound)	6.71 ± 0.07	2.20 ± 0.07
γ-CD (bound)	29.80 ± 0.11	
neutral berubicin (bound)	8.47 ± 0.10	1.12 ± 0.06
HP-β-CD (bound)	29.52 ± 0.31	
protonated berubicin (bound)	8.90 ± 0.07	1.38 ± 0.03
HP-β-CD (bound)	29.05 ± 0.21	

a The values reported in parentheses are obtained from other published studies.

b HB_mol–wat_ corresponds to the hydrogen bonds between the molecule (mol) of the first column with water. HB_ber–CD_ corresponds to the hydrogen bonds between berubicin and cyclodextrin in each case.

As for the thermodynamic description of the berubicin–cyclodextrin complexes, the binding Gibbs energy is estimated by employing the LIE method, described by eq 1. The results are listed in Table 7 in which Δ*G*_bind_, along with the corresponding statistical error, is given for all cyclodextrin complexes taken into consideration in this article. It is concluded that in all cases the binding Gibbs energy is negative, verifying that the formation of the noncovalent complexes is a spontaneous process. More precisely, the Δ*G*_bind_ values range between −19.1 and −23.8 kJ/mol. To the best of our knowledge, there do not exist reported values in the literature for the binding Gibbs energies of berubicin with either the cyclodextrins of this article or other cyclodextrin molecules. In view of this, we compare the above-mentioned results of our thermodynamic analysis with those of ref (15) which concerns complexes of doxorubicin with both γ-CD and HP-β-CD. Δ*G*_bind_ values for this drug are provided in the third column of Table 7 and lie in the range between −19.8 and −24.9 kJ/mol that is in agreement with that of berubicin reported in Table 7. So, we conclude that the berubicin’s substitution in the aglycone does not interfere significantly with its ability to enter into cyclodextrin’s cavity. The validity of the binding Gibbs energy values in the doxorubicin–cyclodextrin case in ref (15) is confirmed by experimental results, reported therein for both cyclodextrins.

**Table 7:** Binding Gibbs Energies for the Various Berubicin–Cyclodextrin Complexes Considered Herein^a^

supramolecule	Δ*G*_bind_ [kJ/mol] (drug: berubicin)	Δ*G*_bind_ [kJ/mol] (drug: doxorubicin)
neutral drug – γ-CD	–19.1 ± 0.8	–19.8 ± 0.4
protonated drug – γ-CD	–23.8 ± 1.2	–21.2 ± 0.8
neutral drug – HP-β-CD	–22.1 ± 0.7	–20.2 ± 3.8
protonated drug – HP-β-CD	–23.7 ± 2.6	–24.9 ± 0.8

a The values reported in the third column were taken from ref (15).

## 4 Conclusions

This study deepens our understanding of the interactions between berubicin–DNA and berubicin–cyclodextrins with the drug being simulated in the protonated as well as in the neutral state. Berubicin at pH values above 7 is principally found in the protonated state, but the study of its neutral state as well helps us better comprehend the role of drug charge in the formed complexes. As far as the berubicin–DNA complexes are concerned, this *in silico* study manifests that berubicin is a strong intercalator with the anthraquinone group being inserted in the intercalation sites of both DNA sequences considered herein, a finding that is in full agreement with published articles concerning anthracyclines that are employed as anticancer drugs. Moreover, the minor groove of DNA contributes significantly to the further stabilization of the noncovalent complexes in the case of the protonated berubicin. In the latter, the role of the glycosidic bond is highlighted in terms of the related dihedral angle, comparing and contrasting the bound and unbound state of the drug. The stabilization of the complexes is also owed to the development of a hydrogen bond network in which the anchor and sugar moiety primarily participate, while secondarily, the anthraquinone group contributes as well. The binding Gibbs energy of berubicin into DNA confirms the spontaneity of the process. Furthermore, the calculated values in the case of the protonated berubicin is markedly more negative (≃ −65 kJ/mol) than those of other anthracyclines, such as doxorubicin and daunorubicin. Partitioning the binding Gibbs energy into its entropic and enthalpic contributions reveals that the intercalation of berubicin is enthalpy-driven, as was expected for an intercalation process. Also, the desolvation Gibbs energy is estimated and similarly partitioned into its enthalpic and entropic contributions, in order to quantify how difficult it is to break the solutes’ solvation shells upon complexation. The positive desolvation entropies and enthalpies, as well as Gibbs energies, confirm the hydrophobicity of the berubicin–DNA binding process. The reasonable results obtained from the proposed thermodynamic treatment of the desolvation are promising for future application to other binding scenarios, investigating in detail the role of the solvent.

In addition to DNA, berubicin forms spontaneously noncovalent complexes with both HP-β-CD and γ-CD. MD simulations are carried out to investigate whether cyclodextrins may be a stable carrier for drug delivery purposes. Indeed, in all cases considered herein, the formation of the noncovalent complexes takes place spontaneously. More specifically, berubicin binds to the internal hollow part of the cyclodextrins, namely, the lipophilic cavity, exhibiting negative binding Gibbs energies, which are similar to those of doxorubicin.^15^ In addition to the hydrophobic interactions, berubicin forms a hydrogen bond network in the bound state in which both the cyclodextrin and water molecules participate. It is noteworthy that, upon complexation with cyclodextrins, common characteristics with other anthracyclines and lipophilic drugs are exhibited, such as a displacement of trapped water molecules from the cyclodextrin cavity, an increase in the total SASA that has as a consequence the improvement of the drug’s water solubility, and a negative Δ*S*ASA which indicates the hydrophobic character of the complexes. Due to the bigger cavity size of γ-CD compared to that of HP-β-CD, berubicin fits more effectively in the former cyclodextrin.

In conclusion, our results indicate that berubicin forms stronger intercalation complexes with DNA in comparison with daunorubicin and doxorubicin. This is mainly attributed to its higher hydrophobicity. This predicted relative effectiveness may be related to the experimental finding that berubicin is an anthracycline with enhanced cytotoxicity.^132^ Despite its effective interaction with the target, hydrophobicity and other factors make the drug exhibit pharmacokinetic issues with its administration, such as low solubility, instability, and dimerization. We find that these problems can be suppressed by using a berubicin–cyclodextrin complex, instead of unbound berubicin, due to the higher stability and solubility of the complex. As far as the role of the net charge at berubicin is concerned, we gain physicochemical insights about the nature of the driving forces of the intercalation process as we mentioned. Besides that, the knowledge of the contribution of net charge can be used in drug design of novel anthracyclines with improved properties, as we expect a charged anthracycline to be significantly more potent than an uncharged one.

## Supporting Information Available

Additional theoretical and computational information and technical details about molecular docking, DDM, desolvation thermodynamics, convergence checks, simulated annealing, equilibration and production runs, as well as images from docking runs; RMSD plots of the DNA sequences, studied herein, alone in water; images depicting the relative position of berubicin’s amino group in γ-CD complexes for neutral and protonated states of the former; and results from the decomposition of the binding Gibbs energies of berubicin–DNA complexes into their enthalpic and entropic parts (PDF)
