# Supplementary material for: Interactions of a Novel Anthracycline with Oligonucleotide DNA and Cyclodextrins in an Aqueous Environment
Source: J Phys Chem B. 2024 Jun 20;128(26):6291–307. doi: 10.1021/acs.jpcb.4c02213 (PMC11228990; doi:10.1021/acs.jpcb.4c02213)
Supplement: Supplementary file 1 — jp4c02213_si_001.pdf [file jp4c02213_si_001.pdf]

SUPPORTING INFORMATION

On the Interactions of a Novel Anthracycline with Oligonucleotide DNA and Cyclodextrins in Aqueous Environment

Georgios Mikaelian,[(a)] *Grigorios Megariotis,*[(a,b)(*)] *and Doros N. Theodorou*[(a)]

[(a)] School of Chemical Engineering, National Technical University of Athens (NTUA), 9 Heroon Polytechniou Street, Zografou Campus, Athens, GR, 15780, Greece

[(b)] School of Engineering, Department of Mineral Resources Engineering, University of Western Macedonia, 50100, Kozani, Greece.

[(*)] gregm@mail.ntua.gr

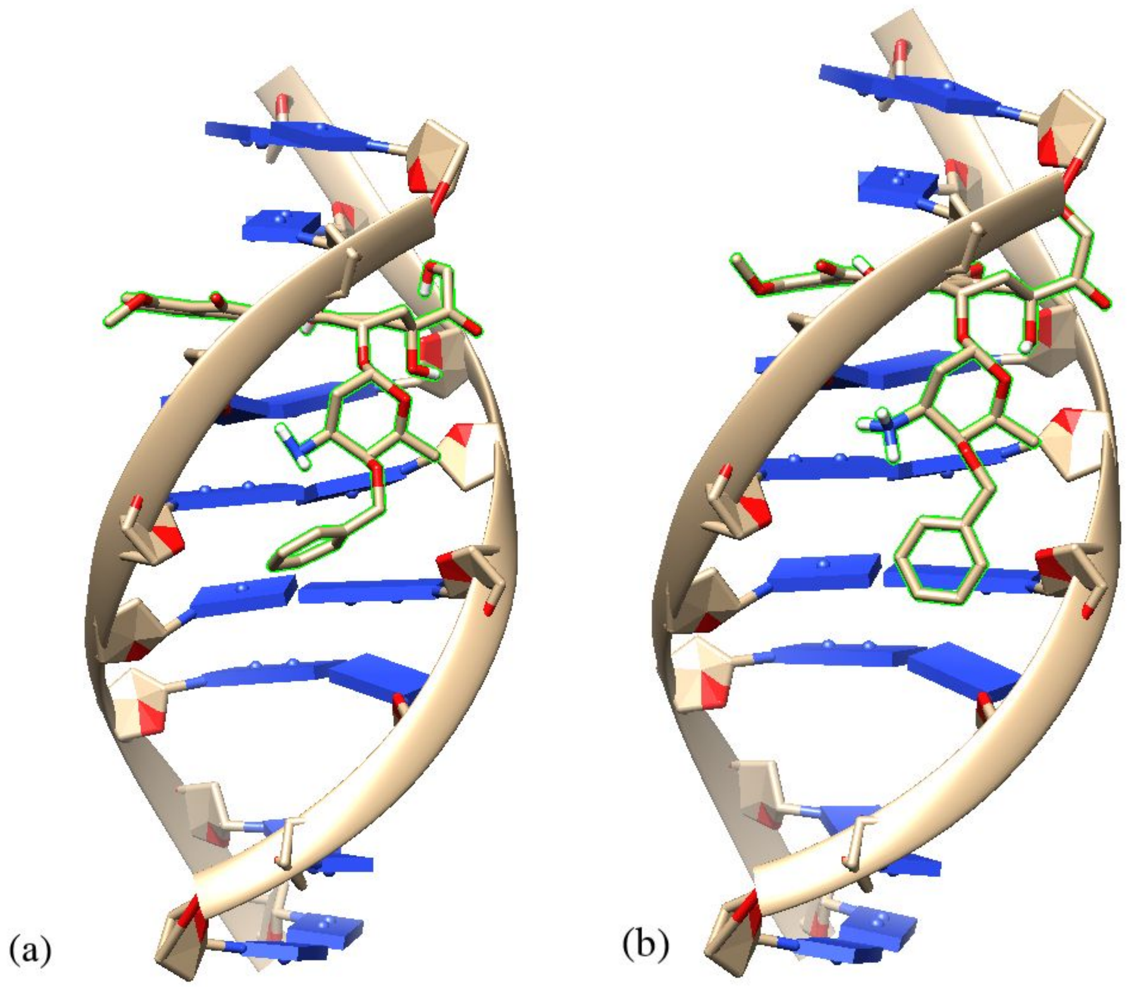

(a)
(b)

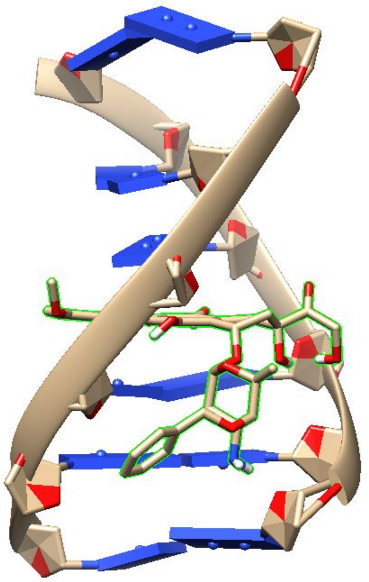

(c)

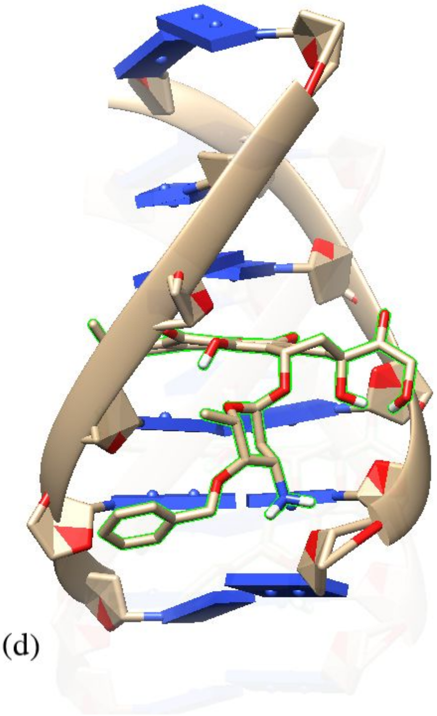

(d)

Figure S1. Graphical representations of berubicin bound in DNA as an intercalating agent obtained from the docking runs: (a): neutral berubicin – 1AL9, (b): protonated berubicin – 1AL9, (c): neutral berubicin – 1AMD and (d): protonated berubicin – 1AMD. It is worth noticing the open second intercalation site existing in every structure, along with the occupied one.

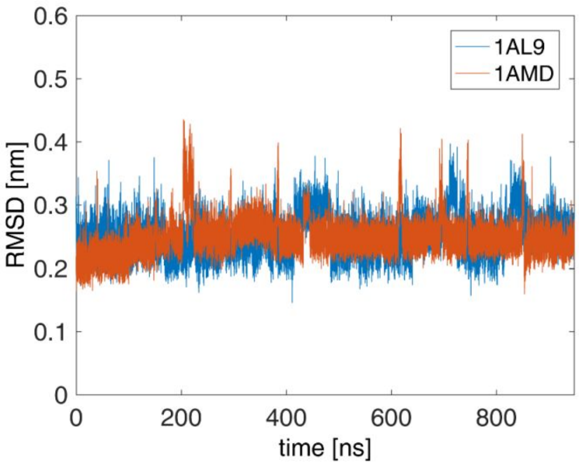

Figure S2. RMSD plots of 1AL9 and 1AMD in the unbound state in water.

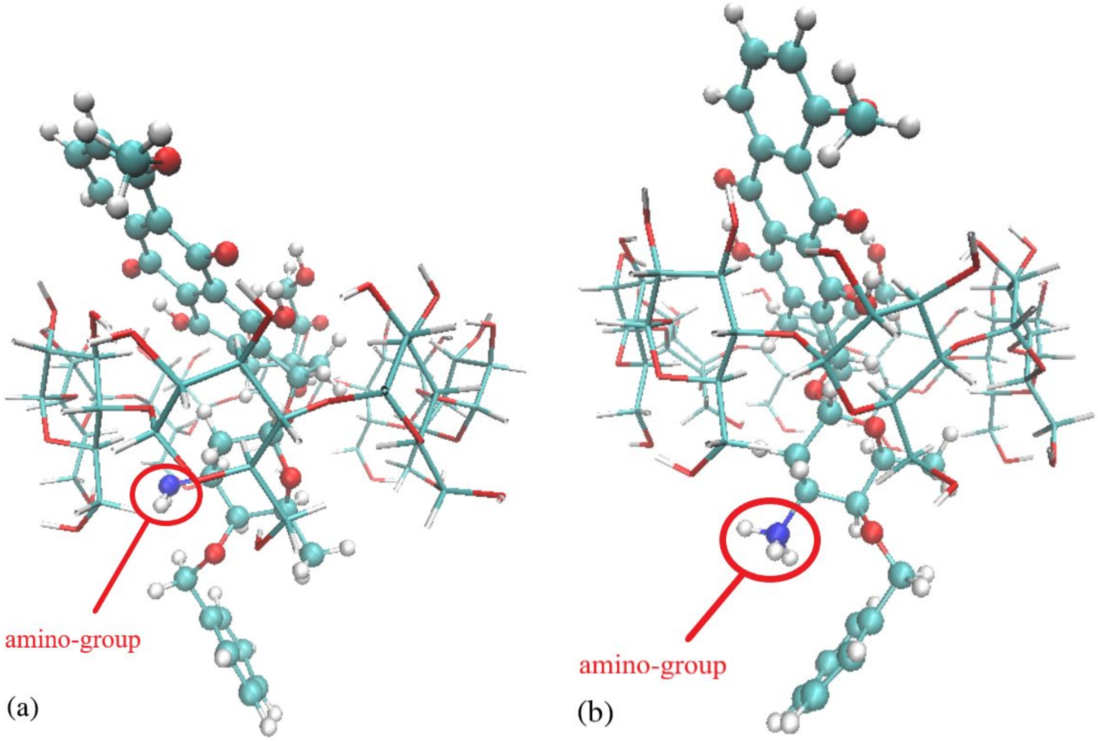

Figure S3. Amino-group's position relative to the γ-CD cavity in the former's (a) neutral and (b) protonated state.

S1. DOCKING SIMULATIONS

The molecular docking performed using the AutoDock program. The blind docking uses a grid box which is as big as the whole DNA molecule, in order to investigate all the possible binding sites. The grid spacing is 0.0375 nm and the average grid box dimensions are 2.3×2.7×3.7 nm^3 for 1AL9 and 2.2×2.4×2.7 nm^3 for 1AMD. The docking algorithm is the Lamarckian Genetic

Algorithm (LGA).[S1] A total of 2.5×10^6 energy evaluations are used in 50 independent runs and the Genetic Algorithm (GA) population size is 300. Regarding the specific-site docking, the grid boxes are smaller, with the same grid spacing. The average grid box dimensions are $2.3 \times 2.4 \times 1.9$ nm^3 and $2.3 \times 2.3 \times 2.1$ nm^3 for the two equivalent 1AL9 intercalation sites and $2.2 \times 2.3 \times 1.7$ nm^3 and $2.4 \times 2.4 \times 1.5$ nm^3 for the median and terminal intercalation sites of 1AMD, respectively. The docking algorithm is LGA, but the searching should be more detailed, so the energy evaluations are 2.0×10^7 and the GA population size is 150 in 50 independent runs. In all the cases, the charge models in use are Gasteiger for berubicin and Kollman for DNA.

S2. TECHNICAL DETAILS ABOUT THE DOUBLE DECOUPLING METHOD

Equation 2 of the article implements DDM, which is schematically described by the thermodynamic cycle of Figure 3 of the article. Technical details and explanations are given in ref 60, so here they will be briefly discussed. For the first term on the right-hand side of eq 2, $\Delta G_{1\rightarrow3}$, 1 μs of molecular dynamics is conducted on all the berubicin – DNA systems in their bound states in water adding a harmonic force constant between one atom on the berubicin and one atom on the DNA with a harmonic constant, k_r, equal to 1000 kJ/mol/nm^2. Then, the free energy, accompanying the switching on of the harmonic distance restraint is given by the Zwanzig free energy perturbation equation:[S2] $\Delta G_{1\rightarrow3} = k_\mathrm{B}T \cdot \left\langle \exp\left(\frac{V_\mathrm{res}}{k_\mathrm{B}T}\right) \right\rangle_\mathrm{res}$, where k_B is the Boltzmann constant, V_res is the harmonic biasing potential and the mean value is extracted throughout the biased simulation. Next, $-\Delta G_{4\rightarrow5}$ is the free energy associated with switching on the harmonic

constant in the gas phase (the interactions of the ligand are turned off) at standard concentration (1 M), in order to properly obtain the binding Gibbs energy at the standard state. This term is estimated theoretically as:[59,60,S3] $-\Delta G_{4\rightarrow 5} = -\, k_{\mathrm{B}}T \cdot \ln\left[\frac{1}{v_0} \cdot \left(\frac{2\pi k_{\mathrm{B}}T}{k_{\mathrm{r}}}\right)^{3/2}\right]$, where v_0 is the volume corresponding to the solute's standard concentration. Last, the terms $\Delta G_{3\rightarrow 4}$ and $\Delta G_{5\rightarrow 2}$ are both extracted alchemically by turning off gradually the electrostatic interactions of the ligand first and then the Lennard-Jones ones. Twenty λ values are used to reach gradually from 0 to 1 with a constant step of 0.05 for both the electrostatic and Lennard-Jones decouplings. In each one, 60 ns of stochastic MD are conducted with the inverse friction constant being 0.2 ps. Thus, $\Delta G_{3\rightarrow 4}$ and $-\Delta G_{5\rightarrow 2}$ are extracted by adding their decoupling free energies from the electrostatic and Lennard-Jones contributions, both of them calculated numerically as $\int_0^1 \left\langle \frac{\partial \mathcal{H}}{\partial \lambda} \right\rangle_\lambda \mathrm{d}\lambda$ in the context of the thermodynamic integration approach,[S4] where $\mathcal{H}$ is the Hamiltonian function. The separation of these terms into their electrostatic and Lennard-Jones contributions gives us the possibility to decompose the total binding Gibbs energy into the electrostatic and van der Waals contributions by combining the electrostatic and Lennard-Jones parts, respectively, of $\Delta G_{3\rightarrow 4}$ and $\Delta G_{5\rightarrow 2}$ (see Table S1). A last comment is about eq 3 of the article that adds a correction term to the binding Gibbs energy in the protonated berubicin cases. This accounts for the periodicity-induced net-charge interactions, periodicity-induced undersolvation, residual integrated potential effects and discrete solvent effects.[61] The residual integrated potential effects are estimated by evaluating the Poisson-Boltzmann equation via the APBS (version 3.4.1) web service.[S5] The theoretical and

technical details of the electrostatic correction, as well as the procedure that is followed to evaluate them, may be found in ref 61.

Table S1. Van der Waals and electrostatic contributions to the binding Gibbs energies.[a]

	Neutral berubicin – 1AL9	Protonated berubicin – 1AL9	Protonated berubicin – 1AMD
$-\Delta G^{\mathrm{el}}_{3\to4}$ [kJ/mol]	–159.2 ± 0.4	–286.3 ± 1.2	–286.2 ± 5.2
$-\Delta G^{\mathrm{L}J}_{3\to4}$ [kJ/mol]	–9.9 ± 2.6	–14.6 ± 4.2	–19.4 ± 0.7
$\Delta G^{\mathrm{el}}_{5\to2}$ [kJ/mol]	–171.6 ± 0.3	–259.4 ± 0.2	–259.4 ± 0.2
$\Delta G^{\mathrm{L}J}_{5\to2}$ [kJ/mol]	+38.8 ± 1.6	+36.9 ± 1.1	+36.9 ± 1.1
$\Delta\Delta G^{\mathrm{corr}}$ [kJ/mol]	–	+0.53 ± 0.03	+0.66 ± 0.03
ΔG_{el} [kJ/mol][a]	+12.4 ± 0.4	–26.4 ± 1.2	–26.1 ± 5.2
ΔG_{vdW} [kJ/mol][a]	–48.7 ± 3.1	–51.5 ± 4.3	–56.3 ± 1.3

[a]The ΔG_{el} and ΔG_{vdW} are calculated as follows; $\Delta G_{\mathrm{el}} = -\,\Delta G_{3\rightarrow 4}^{\mathrm{el}} - \Delta G_{5\rightarrow 2}^{\mathrm{el}} + \Delta\Delta G^{\mathrm{corr}}$ and $\Delta G_{\mathrm{vdW}} = -\,\Delta G_{3\rightarrow 4}^{\mathrm{LJ}} - \Delta G_{5\rightarrow 2}^{\mathrm{LJ}}$. This decomposition allows us to rewrite the binding free energies as $\Delta G_{\mathrm{bind}} = \Delta G_{\mathrm{vdW}} + \Delta G_{\mathrm{el}} - \Delta G_{1\rightarrow 3} - \Delta G_{4\rightarrow 5}$ beginning from eqs 2 and 3 of the article.

S3. DETAILS ABOUT DESOLVATION GIBBS ENERGY AND THERMODYNAMICS

We are interested in extracting the desolvation thermodynamics in the berubicin – DNA complexes. To this end, a thermodynamic cycle, similar to those in refs 105 and S6 and in most continuous-solvent approaches extracting the Gibbs energy, such as MM-PBSA and MM-GBSA,[S7,S8] is constructed in Figure S4. This thermodynamic cycle is summarized by the next equation:

$$\Delta G_{1\rightarrow 2} = \Delta G_{3\rightarrow 4} + (\Delta G_{1\rightarrow 3} + \Delta G_{4\rightarrow 2}) \qquad \text{(S1)}$$

The left-hand side of eq S1 is the binding Gibbs energy itself, ΔG_{bind}. $\Delta G_{3\rightarrow 4}$ corresponds to the *in-vacuo* binding Gibbs energy, $\Delta G_{\mathrm{drug-DNA}}$. $(\Delta G_{1\rightarrow 3} + \Delta G_{4\rightarrow 2})$ is the desolvation Gibbs energy, ΔG_{des}, since it takes into account the desolvation of both the solute molecules as well as the solvation of the formed complex. With these definitions, eq S1 gives:

$$\Delta G_{\mathrm{des}} = \Delta G_{\mathrm{bind}} - \Delta G_{\mathrm{drug-DNA}} \qquad \text{(S2)}$$

Of course, the same thermodynamic analysis is valid for any state function, such as enthalpy and entropy. Therefore, similar equations as eq S2 hold for these functions by replacing the Gibbs energy, G, with enthalpy, H, and entropy, S. These are eqs 6 of the article and are used in current desolvation thermodynamics. DDM and SBM provide all the binding parameters, i.e. ΔG_{bind}, ΔH_{bind} and ΔS_{bind}. $\Delta H_{\mathrm{drug-DNA}}$ is easily obtainable by computing the direct intermolecular and intramolecular interactions, involving only berubicin and DNA in the unbound and bound states

(in a similar manner to the molecular mechanics term of the MM-PBSA and MM-GBSA methods) and then taking the differences of the form $\langle V\rangle_{\mathrm{AB}} - (\langle V\rangle_{\mathrm{A}} + \langle V\rangle_{\mathrm{B}})$, which are assumed to be approximately equal to $H_{\mathrm{AB}} - (H_{\mathrm{A}} + H_{\mathrm{B}})$, where $\langle V\rangle$ stands for the intramolecular and intermolecular potential energy interactions only between the solutes. $\Delta S_{\mathrm{drug-DNA}}$ is estimated by the WSAS model, being employed in both the unbound and bound states and then taking differences of the form $S_{\mathrm{AB}} - (S_{\mathrm{A}} + S_{\mathrm{B}})$. The indices A, B and AB refer to the solvated simulations containing only the receptor, ligand and complex, respectively. Having these, it is possible to obtain the desolvation parameters by applying the eqs 5, 6 and S2.

This approach of extracting desolvation Gibbs energies has similarities with the corresponding desolvation parts in MM-PBSA and MM-GBSA approaches, but it seems to overpass some of the main disadvantages of them. Firstly, the accuracy of these methods is highly dependent to the parameters in use to the solvation approaches,[S8] such as solutes' dielectric constant, whereas our approach does not need any extra parametrization. Also, Poisson-Boltzmann approach gives high uncertainties for highly charged or polar compounds.[S8,S9] The use of an implicit solvent introduces errors regarding possible water molecules which contribute to the binding,[S8-S10] either by their existence or dislocation. Our approach keeps the solvent explicit. Last, the formulation, followed herein, allows the decomposition of the desolvation Gibbs energy into its enthalpic and entropic parts. Although this method overpasses some of the disadvantages of the above-mentioned ones, it is more time-consuming, due to the two alchemical decouplings in DDM. Another rigorous method that could be used to approach the desolvation Gibbs energy is the direct extraction of $(\Delta G_{1\rightarrow 3} + \Delta G_{4\rightarrow 2})$ via three alchemical runs. $\Delta G_{4\rightarrow 2}$ is the coupling Gibbs energy (turning on the

interactions) of the whole complex from vacuum to water and $\Delta G_{1\rightarrow 3}$ is the sum of the decoupling (turning off the interactions) Gibbs energies of the ligand and receptor from water to vacuum. But this approach would be much more time-consuming. A last comment on these is that the formulation, showed herein, allows any approximation of the involving quantities, ΔG_{bind} and $\Delta G_{\mathrm{drug-DNA}}$, and their enthalpic and entropic parts, offering flexibility to the method. In conclusion, the method, showed herein, seems to combine well accuracy, flexibility and efficiency.

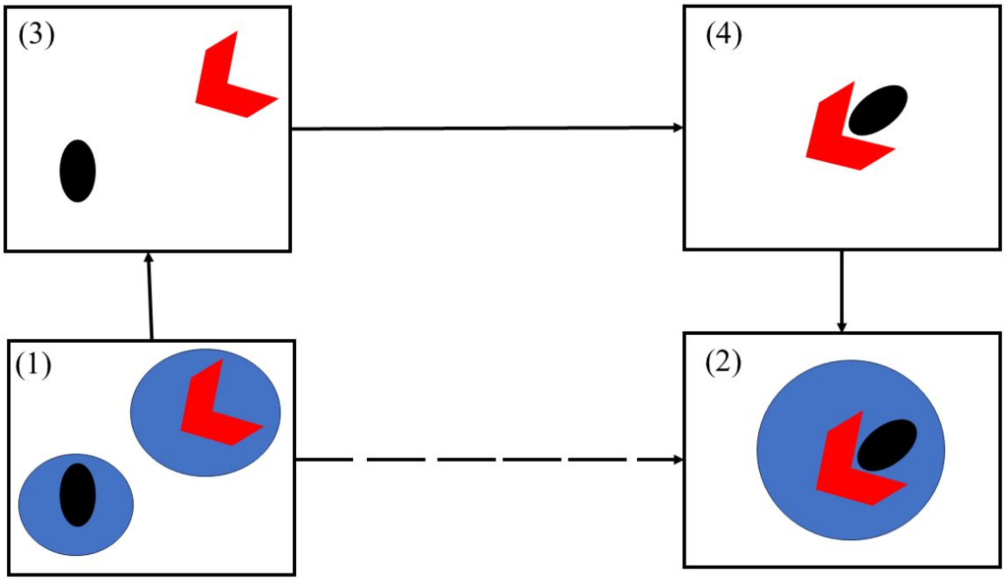

Figure S4. Thermodynamic cycle employed for the calculation of the desolvation Gibbs energies. The red and black objects stand for the receptor and the ligand, respectively. When the objects are inside the blue circle, then they are solvated, otherwise they are desolvated (the solvent is away). State (1) refers to the unbound state in which both the solutes are solvated in solution, whereas state (2) refers to the solvated formed complex between them in solution. State (3) refers to the unbound desolvated state in which the molecules are far from each other *in-vacuo* and state (4) refers to the bound desolvated complex. The materialization of this thermodynamic cycle is executed via eq S1, the description of which is given in Sections 2 and S3.

S4. CONVERGENCE CHECK OF THE MAIN ROTATIONAL AND TRANSLATIONAL DEGREES OF FREEDOM

An important aspect of MD simulations is the convergence of the rotational and translational degrees of freedom of the studied systems, because their convergence indicate that the phase space is properly sampled. Regarding the rotational degrees of freedom, the three principal axes of inertia of the solutes of all the unbound systems (neutral berubicin, protonated berubicin, γ-CD, HP-β-CD, 1AL9 and 1AMD) are computed as functions of the simulation time. Then, the autocorrelation functions of these axes are estimated as the mean values of the second-order Legendre polynomial of the cosine of the angle between the same axis at two different times:

$$C(t) = \frac{P_2\left[\langle \cos[\theta(t)] \rangle\right]}{P_2\left[\langle \cos[\theta(0)] \rangle\right]}$$

where $P_2(x) = \frac{1}{2}(3x^2 - 1)$ is the second-order Legendre polynomial and $\cos[\theta(t)] = \hat{\boldsymbol{p}}(t) \cdot \hat{\boldsymbol{p}}(0)$ is the angle between the unit vector on the principal axis, $\hat{\boldsymbol{p}}$, at different time-frames with a difference of time t between them. The brackets indicate the mean value from the several time-frames with time-difference of t. The denominator $P_2[\langle \cos[\theta(0)] \rangle]$ normalizes the autocorrelation function. Indicatively, Figure S5 shows this autocorrelation function for the major principal axis in three characteristic cases; protonated berubicin, γ-CD and 1AL9. We see an exponential decay of the autocorrelation functions down to zero, indicating that after some time any memory about the axes' orientation is lost. This randomization of the principal axes' orientations indicates that the main rotational degrees of freedom are properly sampled. To quantify this conclusion, the decorrelation characteristic times are extracted by integrating the autocorrelation functions,[S11] assuming that the decay is exponential. Table S2 contains all the characteristic times. It is seen that all of them are significantly lower than the 1 μs duration of the main MD runs.

As far as the translational degrees of freedom are concerned, the Fickian diffusion of the unbound systems reveals the rate of the convergence of the molecules' COMs. To this end, the mean square displacement (MSD) is extracted as a function of the simulation time. Indicative plots for the same three cases, as in Figure S5, are given in Figure S6. In all the cases, we see a linear region which indicates Fickian diffusion. The slopes of these lines are related with the diffusion coefficients via the Einstein relation, $D = \frac{1}{6} \cdot \frac{\mathrm{MSD}}{t}$. From this, we estimate the diffusion lag time which should again be significantly lower than the simulation time to ensure that the Fickian diffusion is present and the molecules have enough time to sample most of their translational degrees of freedom. This is computed as $t_L = \frac{L^2}{6D}$, where L is the simulation box length which plays the role of the characteristic diffusion length of the simulations. Table S3 contains all the lag times, as well as the coefficient of determination of the linear regression between MSD and t, R^2.

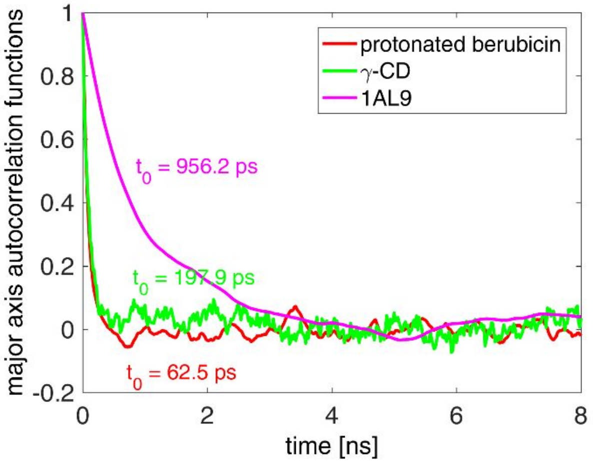

Figure S5. Rotational autocorrelation functions of the major principal axes of inertia in three indicative unbound cases. t_0 is the characteristic correlation time (see the text herein for more information).

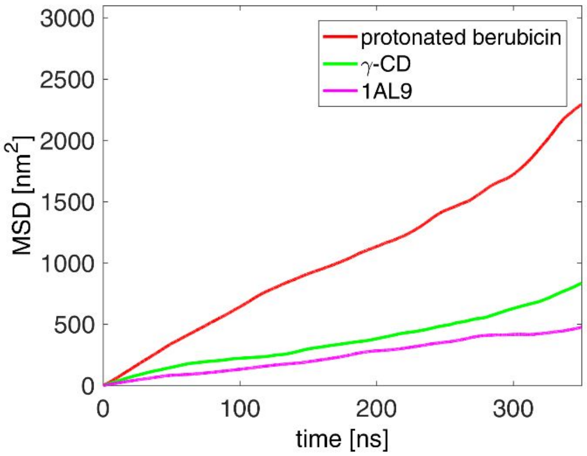

Figure S6. Mean square displacement of the same three indicative unbound cases, as in Figure S5.

Table S2. Correlation times for every unbound case. The correlation times are referred to the major (top value), middle (middle value) and minor (bottom value) principal axis of inertia. The time units are picoseconds (ps).

Neutral berubicin	Protonated berubicin	γ-CD	HP-β-CD	1AL9	1AMD

91.2 $\pm$ 0.1	62.5 $\pm$ 0.1	197.9 $\pm$ 0.1	204.0 $\pm$ 0.1	956.2 $\pm$ 0.1	782.0 $\pm$ 0.1
51.7 $\pm$ 0.1	66.3 $\pm$ 0.1	106.6 $\pm$ 0.1	188.8 $\pm$ 0.1	765.5 $\pm$ 0.1	769.2 $\pm$ 0.1
54.9 $\pm$ 0.1	61.9 $\pm$ 0.1	128.9 $\pm$ 0.1	182.9 $\pm$ 0.1	782.4 $\pm$ 0.1	706.0 $\pm$ 0.1

Table S3. Lag times and the coefficients of determination of the linear regression between MSD and t for every unbound case.

System	Lag time (ns)	R^2
Neutral berubicin	3.36 $\pm$ 0.01	0.99
Protonated berubicin	3.75 $\pm$ 0.01	0.99
γ-CD	13.54 $\pm$ 0.01	0.96
HP-β-CD	10.57 $\pm$ 0.01	0.95
1AL9	46.78 $\pm$ 0.01	0.99
1AMD	20.48 $\pm$ 0.01	0.99

S5. CONFORMATIONAL CONVERGENCE OF THE SIMULATED SYSTEMS

Unbiased MD offers us the opportunity to explore the system's phase space, according to the physical laws and without biases. But often they lack of convergence or non-ergodicity, due to high-energy barriers at the potential energies which result in trapping the studied systems in specific local minima of the potential energy. Therefore, there is a need to validate whether this is not happening at the present simulations and the ergodicity is not violated. A well-known strategy to this kind of simulations is the Simulated Annealing (SA) and is generally used in the literature.[S12,S13] SA consists of simulations starting from a high temperature and gradually linearly decreasing it with time. The high temperature, in combination with a slow annealing, gives to the system the ability to overpass certain potential energy local minima, due to the higher thermal energy they possess.

The SA simulation is conducted starting from a temperature of 345 K and it gradually decreases with a rate of 1.5 K per 20 ns down to 310 K, which is the temperature of the productive MD runs, and then it is kept constant at 310 K for the rest time. Therefore, the first 460 ns of the SA simulation the temperature is decreasing and the rest has become an ordinary *NPT* simulation. The initial temperature should not be much higher than the selected one in order not to exceed the melting temperature and face undesired conformational changes to the oligonucleotide, as the melting temperature is in the region of 340 – 350 K.[S14,S15] The main output of this calculation is the RMSD (of the same heavy atoms and the same reference structure for proper comparisons with the constant-temperature *NPT* simulations, conducted herein), depicted in Figure S7. It is about the neutral berubicin – 1AL9 case. We see that the RMSD has a significant fluctuation at the beginning of the SA simulation, but then it stabilizes at low values, due to the decrease of the

temperature and consequently of the molecular mobility. It is observed that the converged values of RMSD are very similar with the ones obtained from the productive MD simulations of this study (Figures 4 and S2).

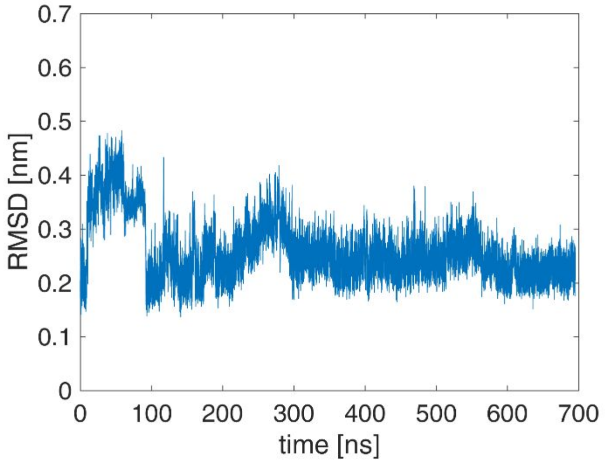

Figure S7. RMSD plot of the neutral berubicin – 1AL9 during the SA run (see text for more information). It is reminded that the annealing process extends from the beginning (345 K) up to 460 ns (310 K). From 460 ns on, the simulation samples the ordinary *NPT* statistical ensemble.

S6. PROTOCOLS FOR THE EQUILIBRATION AND PRODUCTIVE MD RUNS

Prior to the beginning of the production simulations, the systems are properly equilibrated. The equilibration protocol for the cyclodextrin-based complexes is exactly the same as in ref 35 (Section 2 therein), which investigates a complex formed between β-cyclodextrin and fluoxetine, a widely-prescribed drug acting on the central nervous system. The production stage of all drug – cyclodextrin simulations is 1 μs long. Regarding the berubicin – DNA systems, the equilibration protocol, which is applied in Amber 14 making use of the SANDER module, is as follows. First,

an energy minimization step is applied to the solvated systems, which comprises 10000 iterations, using positional restraints on all DNA and drug atoms with a harmonic constant of 1000 kcal/mol/nm^2. Next, a new energy minimization step of 10000 iterations is carried out in absence of these positional restraints. After this two-step energy minimization, the systems are heated to 310 K from 0 K by conducting MD for 200 ps under constant volume with the use of a Langevin thermostat[S16] and a collision frequency equal to 1.0 ps^{-1} and under the same positional restraints as imposed during the first minimization step. Then, a MD simulation of 2 ns is conducted at the temperature of 310 K and pressure of 1 bar, without the restraints and by applying the Berendsen barostat[S17] with the pressure relaxation time being 1.0 ps. The last two simulations are conducted employing an integration time step of 2 fs with all bonds containing hydrogen atoms constrained to their equilibrium length by use of the SHAKE algorithm.[S18] Subsequently, a MD simulation of 25 ns is carried out at the same temperature and pressure in GROMACS, using a time step of 2 fs and the LINCS algorithm[S19] for constraining the bonds containing hydrogen atoms, and without positional restraints.

The production MD simulations are carried out in the *NPT* statistical ensemble and particularly at the temperature of 310 K, using the Nosé-Hoover thermostat[S20,S21] with a time constant of 1.0 ps, and at a pressure of 1 bar, using the Parrinello-Rahman barostat[S22] with a time constant of 1.0 ps. The numerical integration time step for the equations of motion, is set to 2.0 fs, whereas all bonds involving hydrogen atoms are constrained to their equilibrium length employing the LINCS algorithm. The long-range electrostatics is treated with the well-known particle mesh Ewald method,[S23] while the Lennard-Jones interactions exerted between the atoms are calculated with a

cutoff radius of 1.0 nm. In the latter case, the corresponding forces are smoothly switched to zero in the range between 0.9 nm and the cut-off distance, 1.0 nm, by using the force-switch option which is available in the GROMACS package. In addition, long-range energy and pressure correction terms are applied due to the truncation of the non-bonded potentials. Regarding the molecular visualization, in addition to VMD, most of the berubicin – DNA complexes are visualized with the UCSF Chimera software[S24] in which the DNA strands are represented as ribbons, while the drug is depicted in the united atom representation.

REFERENCES

(S1) Morris, G. M.; Goodsell, D. S.; Halliday, R. S.; Huey, R.; Hart, W. E.; Belew, R. K.; Olson, A. J. Automated docking using a Lamarckian genetic algorithm and an empirical binding free energy function. *J. Comput. Chem.* **1998**, *19* (14), 1639-1662. DOI: 10.1002/(SICI)1096-987X(19981115)19:14%3C1639::AID-JCC10%3E3.0.CO;2-B

(S2) Zwanzig, R. W. High-temperature equation of state by a perturbation method. I. Nonpolar gases. *J. Chem. Phys.* **1954**, *22* (8), 1420-1426. DOI: 10.1063/1.1740409

(S3) Deng, N.; Wickstrom, L.; Cieplak, P.; Lin, C.; Yang, D. Resolving the ligand-binding specificity in c-MYC G-quadruplex DNA: absolute binding free energy calculations and SPR experiment. *J. Phys. Chem. B* **2017**, *121* (46), 10484-10497. DOI: 10.1021/acs.jpcb.7b09406

(S4) Kirkwood, J. G. Statistical mechanics of fluid mixtures. *J. Chem. Phys.* **1935**, *3* (5), 300-313. DOI: 10.1063/1.1749657

(S5) Jurrus, E.; Engel, D.; Star, K.; Monson, K.; Brandi, J.; Felberg, L. E.; Brookes, D. H.; Wilson, L.; Chen, J.; Liles, K.; et al. Improvements to the APBS biomolecular solvation software suite. *Protein Sci.* **2018**, *27* (1), 112-128. DOI: 10.1002/pro.3280

(S6) Chervenak, M. C.; Toone, E. J. A direct measure of the contribution of solvent reorganization to the enthalpy of binding. *J. Am. Chem. Soc.* **1994**, *116* (23), 10533-10539. DOI: 10.1021/ja00102a021

(S7) King, E.; Aitchison, E.; Li, H.; Luo, R. Recent developments in free energy calculations for drug discovery. *Front. Mol. Biosci.* **2021**, *8*, 712085. DOI: 10.3389/fmolb.2021.712085

(S8) Homeyer, N.; Gohlke, H. Free energy calculations by the molecular mechanics Poisson−Boltzmann surface area method. *Mol. Inf.* **2012**, *31* (2), 114-122. DOI: 10.1002/minf.201100135

(S9) Wang, E.; Sun, H.; Wang, J.; Wang, Z.; Liu, H.; Zhang, J. Z.; Hou, T. End-point binding free energy calculation with MM/PBSA and MM/GBSA: strategies and applications in drug design. *Chem. Rev.* **2019**, *119* (16), 9478-9508. DOI: 10.1021/acs.chemrev.9b00055

(S10) Young, T.; Abel, R.; Kim, B.; Berne, B. J.; Friesner, R. A. (2007). Motifs for molecular recognition exploiting hydrophobic enclosure in protein–ligand binding. *Proc. Natl. Acad. Sci.* **2007**, *104* (3), 808-813. DOI: 10.1073/pnas.0610202104

(S11) Harmandaris, V. A.; Mavrantzas, V. G.; Theodorou, D. N. Atomistic molecular dynamics simulation of polydisperse linear polyethylene melts. *Macromolecules* **1998**, *31* (22), 7934-7943. DOI: 10.1021/ma980698p

(S12) Mondal, S.; Samajdar, R. N.; Mukherjee, S.; Bhattacharyya, A. J.; Bagchi, B. Unique features of metformin: a combined experimental, theoretical, and simulation study of its structure, dynamics, and interaction energetics with DNA grooves. *J. Phys. Chem. B* **2018**, *122* (8), 2227-2242. DOI: 10.1021/acs.jpcb.7b11928

(S13) Sgouros, A. P.; Knippenberg, S.; Guillaume, M.; Theodorou, D. N. Multiscale simulations of polyzwitterions in aqueous bulk solutions and brush array configurations. *Soft Matter* **2021**, *17* (48), 10873-10890. DOI: 10.1039/D1SM01255J

(S14) Kannan, S.; Zacharias, M. Folding of a DNA hairpin loop structure in explicit solvent using replica-exchange molecular dynamics simulations. *Biophys. J.* **2007**, *93* (9), 3218-3228. DOI: 10.1529/biophysj.107.108019

(S15) Yoshizawa, S.; Kawai, G.; Watanabe, K.; Miura, K. I.; Hirao, I. GNA trinucleotide loop sequences producing extraordinarily stable DNA minihairpins. *Biochemistry* **1997**, *36* (16), 4761-4767. DOI: 10.1021/bi961738p

(S16) Izaguirre, J. A.; Catarello, D. P.; Wozniak, J. M.; Skeel, R. D. Langevin stabilization of molecular dynamics. *J. Chem. Phys.* **2001**, *114* (5), 2090-2098. DOI: 10.1063/1.1332996

(S17) Berendsen, H. J.; Postma, J. V.; Van Gunsteren, W. F.; DiNola, A. R. H. J.; Haak, J. R. Molecular dynamics with coupling to an external bath. *J. Chem. Phys.* **1984**, *81* (8), 3684-3690. DOI: 10.1063/1.448118

(S18) Ryckaert, J. P.; Ciccotti, G.; Berendsen, H. J. Numerical integration of the cartesian equations of motion of a system with constraints: molecular dynamics of n-alkanes. *J. Comput. Phys.* **1977**, *23* (3), 327-341. DOI: 10.1016/0021-9991(77)90098-5

(S19) Hess, B.; Bekker, H.; Berendsen, H. J.; Fraaije, J. G. LINCS: A linear constraint solver for molecular simulations. *J. Comput. Chem.* **1997**, *18* (12), 1463-1472. DOI: 10.1002/(SICI)1096-987X(199709)18:12%3C1463::AID-JCC4%3E3.0.CO;2-H

(S20) Nosé, S. A molecular dynamics method for simulations in the canonical ensemble. *Mol. Phys.* **1984**, *52* (2), 255-268. DOI: 10.1080/00268978400101201

(S21) Hoover, W. G. Canonical dynamics: Equilibrium phase-space distributions. *Phys. Rev. A* **1985**, *31* (3), 1695. DOI: 10.1103/PhysRevA.31.1695

(S22) Parrinello, M.; Rahman, A. Polymorphic transitions in single crystals: A new molecular dynamics method. *J. Appl. Phys.* **1981**, *52* (12), 7182-7190. DOI: 10.1063/1.328693

(S23) Darden, T.; York, D.; Pedersen, L. Particle mesh Ewald: An N· log (N) method for Ewald sums in large systems. *J. Chem. Phys.* **1993**, *98* (12), 10089-10092. DOI: 10.1063/1.464397

(S24) Pettersen, E. F.; Goddard, T. D.; Huang, C. C.; Couch, G. S.; Greenblatt, D. M.; Meng, E. C.; Ferrin, T. E. UCSF Chimera—a visualization system for exploratory research and analysis. *J. Comput. Chem.* **2004**, *25* (13), 1605-1612. DOI: 10.1002/jcc.20084
